# Sepal Identity of the Pappus and Floral Organ Development in the Common Dandelion (*Taraxacum officinale*; Asteraceae)

**DOI:** 10.3390/plants10081682

**Published:** 2021-08-16

**Authors:** Kitty Vijverberg, Monique Welten, Marjan Kraaij, Bertie Joan van Heuven, Erik Smets, Barbara Gravendeel

**Affiliations:** 1 Evolutionary Ecology, Naturalis Biodiversity Center, Darwinweg 2, 2333 CR Leiden, The Netherlands; monique.welten@naturalis.nl (M.W.); bertiejoan.vanheuven@naturalis.nl (B.J.v.H.); erik.smets@naturalis.nl (E.S.); barbara.gravendeel@naturalis.nl (B.G.); 2 Experimental Plant Ecology, Institute for Water and Wetland Research (IWWR), Radboud University, Heyendaalseweg 135, 6500 GL Nijmegen, The Netherlands; 3 Evolutionary Genetics, Groningen Institute for Evolutionary Life Sciences (GELIFES), University of Groningen, Nijenborgh 7, 9747 AG Groningen, The Netherlands; kraaij.marjan@gmail.com

**Keywords:** *APETALA*1-like, Asteraceae, dandelion (*Taraxacum officinale*, *Tof*), floral development, inferior ovary, pappus, qPCR, sepals

## Abstract

The dry one-seeded fruits (*cypselae*) of the Asteraceae are often crowned with a *pappus*, an appendage of hairs or scales that assists in dispersal. It is generally assumed, but little investigated, that the pappus represents the outer floral whorl where the *sepals* are usually located. We analysed pappus–sepal homology in dandelions using micromorphological and floral gene expression analyses. We show that the pappus initiates from a ring primordium at the base of the corolla, heterochronic to the petals. Pappus parts form from this ring, with those in the *alternipetalaous* position usually being ahead in growth, referring to sepal identity. *Tof-APETALLA*1 expression increased during floret development and was highest in mature pappus. *Tof-PISTILLATA* expression was high and confined to the floral tissues containing the petals and stamens, consistent with expectations for sepals. Apart from the pappus, the dispersal structure of dandelion consists of the upper part of the fruit, the *beak*, which originates from the inner floral whorl. Thus, our results support the homology of the pappus with the sepals, but show that it is highly derived. Together with our floral stage definitions and verified qPCR reference genes, they provide a basis for evolution and development studies in dandelions and possibly other Asteraceae.

## 1. Introduction

We investigated the development of the pappus in dandelion in detail, aiming to develop the use of dispersal capacity for urban evolution studies in plants. The pappus plays an important role in the dispersal of the seeds and fruits of the Asteraceae [1,2], and variation in the pappus may directly lead to differences in dispersal ability [3,4] allowing plants to anticipate changing environments. To measure such changes and determine how quickly they occur in response to changing environments, we should first characterize pappus development and investigate pappus–sepal homology. To this aim, we performed micro-morphological and A- and B-floral gene expression analyses. Confirmation of pappus–sepal homology would imply the validity of changes in associated A- and B- floral gene expression, and in the longer term their underlying genes, in the evolution of dispersal capacity. Along with our characterization of the pappus, the development of the other floral organs was examined and a reference dataset of the floral developmental stages in dandelion prepared. In addition, the underlying vasculature was investigated. Our results serve as a reference for future studies on the development of dandelion florets and evolution of dispersal capacity. 

### 1.1. Evolution of Dispersal Capacity

The dispersal of seeds and fruits is the main opportunity of plants to migrate and herewith a key factor in species distribution, the promotion of genetic connectivity and diversity in plant communities [5], and the colonization of new habitats [6,7]. Dispersal capacity thus has an important impact on patterns of biodiversity [8]. In today’s rapidly changing environments due to human activity, especially as a result of intensified agriculture and urbanization, the dispersal of seeds and fruits may be under pressure by the reduction of suitable habitats [9,10]. Rapid changes in dispersal strategy in response to urbanization have been found in *Crepis sancta* (Asteraceae), which showed a shift towards the production of higher proportions of less dispersive seeds in urban populations within 5–12 generations [11]. This was interpreted as being advantageous because falling close to the mother plant increases the chances of finding the most suitable habitat. It was a first indication for human induced rapid evolutionary change (HIREC) [12,13] in plants. Whether the diversification of plant populations indeed accelerates as a result of human activity, and to what extent, is yet unknown. Because of the vulnerability of dispersal traits to adapt to changing environments, they are excellent candidates for studying HIREC. To this aim, we should first map the dispersal traits morphologically and specify their underlying genetic bases.

### 1.2. Pappus Development in the Asteraceae

One of the most important traits for seed and fruit dispersal in the Asteraceae is the pappus, a scaly, bristly, or hairy appendage that facilitates uptake by wind or animals [14,15]. Fruits of the Asteraceae are dry, indehiscent, and one-seeded, and developed from an *inferior bicarpellary ovary* [16]. Such fruits are called *cypselae* (versus achenes that are formed from a *superior monocarpellary ovary*, which does not occur in Asteraceae). The fruits of most Asteraceae species are crowned by a pappus that assists in their dispersal and may function in protecting the individual flowers and fruits against herbivores [17] and stimulating water uptake to facilitate germination [18]. This dual or triple functionality is viewed as one of the reasons for the large success of Asteraceae, which cover an estimated 25,000–35,000 species [19].

The Asteraceae is the largest eudicot plant family, consisting of twelve subfamilies [20,21] including two large, derived *crown*-groups: the Asteroideae (sunflower-like) and Cichorioideae (lettuce-like). The Asteraceae are well-known because of their flower heads, named *capitula*, which resemble flowers but are highly compressed racemose inflorescences [22]. The capitulum is surrounded by involucral bracts or scales, the phyllaries, which resemble sepals of individual flowers and have a protective function. The true individual flowers, named *florets* in Asteraceae, are placed closely together on a common receptacle [23]. The florets vary among species mainly in terms of the shape of their corolla, which always consists of five petals, and fertility [24]. The major floret types are: (1) *disc*-flowers, in which all the petals are fused into a tube, and most often producing male and female reproductive organs (hermaphrodite); (2) *ray*-flowers, in which three petal lobes are fused into a ray and the remaining two remain rudimentary and female; and (3) *ligulate*-flowers, in which the five petals are fused into a strap-shaped corolla part or ligule while also being hermaphroditic. The disc- and ray-flowers characterize the species of the Asteroideae, whereas the ligulate-flowers are typical of the Chichorioideae. Being *pentamerous* [25], the florets usually contain five stamens of which the filaments are (partly) fused to the corolla [24]; the anthers (*thecae*) are large and often connate forming a tube around the style. The pistil consists of a bicarpellary inferior ovary, a style, and two stigmas. On top of the inferior ovary, a floral nectary is present. The florets lack an outer whorl of sepals named a *calyx*, but often carry a pappus.

As a result of its position on the outside of the florets and at the base of the corolla, the *pappus* is generally thought to be a modified calyx [26]. This is known as the *phyllome* theory [27,28]. However, its exact origin is hardly investigated, and the pappus has also been viewed as having a non-calycine nature [28], partly adopted by [29], for instance the *trichome* theory [27,30]. One of the functions of sepals is the prevention of the developing flowers against damage from external factors, but this might have become (partly) redundant in Asteraceae, since the involucral bracts or scales protect the inflorescence as a whole [24]. If redundant, total loss or the transformation to another function would be obvious and a role in dispersal quite possible. Remains of a sepaloid origin are then expected to be found in the pappus, both morphologically and molecularly. 

### 1.3. The ABC(D)E-Model of Floral Organ Development

The genetic basis underlying floral development and diversification is summarized in the *ABC(D)E-model*, which refers to master regulatory genes involved in the determination of floral organ identity [31,32]. These genes are transcription factors (TFs) [33], usually of the MADS-box type, that function in various combinations, mostly tetramers [34], each with its own specificity. A/E-TFs are involved in the specification of the sepals, A/B/E in the petals, B/C/E in the stamens, and C/E in the carpels, while D-TFs have a role in ovule development and fruit dehiscence. In the model species *Arabidopsis thaliana* (*At*, Brassicaceae; Rosids), in which the ABC(D)E-model was discovered, these genes include the A-genes *APETALA*1 (*AP*1) and *AP*2; B-genes *AP*3 and *PISTILLATA* (*PI*); C-gene *AGAMOUS* (*AG*); D-genes (or C-like, since an *AG*-subclade) *SHATTERPROOF*1 (*SHP*1), *SHP*2, and *SEEDSTICK* (*STK*); and E-genes *SEPALLATA*1 (*SEP*1) to *SEP*4. In other plant species, this model is conserved with modifications [35]. One remarkable difference concerns the functionality of the A-genes, which in Arabidopsis act, apart from in the establishment of sepal and petal identity, on the spatial restriction of C-gene expression to the inner two whorls, where the stamens and carpels are located (whorls 3 and 4), and the establishment of floral meristem identity [36,37]. Homologs of *AP*1 and *AP*2 in other species mainly or solely share the latter functionality, namely the establishment of floral meristem identity [38,39], suggesting that the specification of floral organ identity in the outer two whorls, where sepals and petals are located (whorls 1 and 2), is indirect. The patterning of the C-gene in *Antirrhinum major* (Plantaginaceae; Asterids) and Petunia (Solanaceae; Asterids) was shown to involve regulation by a microRNA instead [40,41]. Apart from Arabidopsis, a unique sepal-specifying gene seems thus lacking. Other notable differences to the basic floral model include gene duplications, gene losses, and sub-functionalization. For instance, a duplication of the B-genes *AP*3 and *PI*, named *DEFICIENS* (*DEF*) and *GLOBOSA* (*GLO*), respectively, in Antirrhinum [42], was found in Petunia in the form of *DEF*/*TOMATO MADS BOX GENE*6 (*TM*6) and GLO1/*GLO*2 [43]. *TM*6 appeared to be the ancestral gene, being lost in Arabidopsis and Antirrhinum, while its functionality was restricted to the determination of stamens and not petals in Petunia [44]. Such duplications of floral homeotic genes drive floral diversification and are thought to be among the factors that led to the rapid radiation and the origin of numerous species in the flowering plants. 

Knowledge of the genetic basis of floral development in Asteraceae is largely based on studies in *Gerbera hybrida*, a cultured ornamental belonging to a relatively small, basal subfamily of the Asteraceae, the Mutisioideae [45]. In Gerbera, *SQUAMOSA*1 (*GSQUA*1) was found as an *AP*1 homolog [46], *GDEF*1–3 and *GGLO*1 as B-homologs [47], *GAGA*1 and *GAGA*2 as C-homologs, and *REGULATOR OF CAPITULUM DEVELOPMENT*1 (*GRCD*1) to *GRCD*8 as a duplicated set of E-homologs, including sub-functionalization [48]. *GSQUA*1 was particularly highly expressed in the inflorescence lateral meristems, vascular bundles of the inflorescence and florets, and in the petals, whereas it lacked expression in the pappus. This indicates that *GSQUA*1 lacks complete A-gene functionality or that the pappus in Gerbera has no sepaloid origin or does not show remnants of such an origin. The additionally identified *GSQUA*2–6, all classified as *AP*1-like [49], also lacked A-gene functionality by showing expression in all or most floral whorls in addition to the vasculature, but lacking clear perianth (whorl 1 and 2) specificity.

More recently, whole genome and transcriptome sequences confirmed the presence and expression of ABC(D)E-homologous genes in other Asteraceae, among them sunflower (*Helianthus annuus*) [50,51] and lettuce (*Lactuca sativa*, *Ls*) [52,53]. Particularly the homologous genes in lettuce, a close relative of dandelion, are of interest for our studies in dandelion. They include the A-gene *LsMADS*55 (~*AP*1); B-genes *LsMADS*3 (~*PI*), *LsMADS*30, and *LsMADS*51; C-gene *LsMADS*69 (~*AG*); and E-genes *LsMADS*4, *LsMADS*6, *LsMADS*14, *LsMADS*23, and *LsMADS*53 [53]. As was found for *GSQUA*1, *LsMADS*55 was highly expressed in the inflorescence meristem, suggesting involvement in the floral transition, and in bracts. However, in contrast to *GSQUA*1, *LsMADS*55 showed specific expression in the pappus and not petals of developing florets in in situ hybridizations. In lettuce, a typical *AP*1 functionality was thus also lacking, but expression in the pappus was confirmed. The *PI*-homolog *LsMAD*S3 showed high expression in the petals and stamens, according to expectations. We used the dandelion homologues of *AtAP*1/ *LsMADS*55 and *AtPI*/*LsMADS*3 in our analysis, hypothesizing their presence and absence of expression in the pappus, respectively, to validate the ABC(D)E-model of the pappus. 

### 1.4. Dandelion

**Figure 1:**
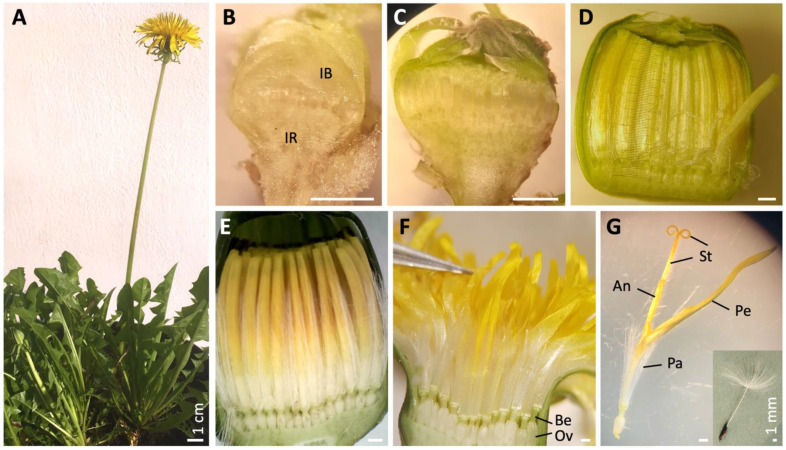
Dandelion plant and capitula at different developmental stages. (**A**) Mature *Taraxacum officinale* plant with one open capitulum (*stage 12*) and one closed bud (*stage 10*), showing the characteristic bare stems, leaf rosettes, and ligulate florets. (**B**) Bud of *stage 2* (Table 1), with floral primordia formed. About the youngest harvestable bud (due to soft tissue, see right under). IR = inflorescence receptacle; IB = involucral bracts. (**C**) Bud of *stage 7 (*stem ~0.2 cm), with all floral organs initiated, corolla lobs covering the top, organs elongating, and pappus length equalling half of the petal length. (**D**) Bud of *stage 9* (stem ~1 cm), showing the elongated upper floral parts, with pigmentation of petals initiated. (**E**) Bud of *Stage 11* (stem ~7 cm), with the corolla still tubular, bud still closed, and the pigmentation of petals finalized. (**F**) Open flower at anthesis, *stage 12*, with corollas forming ligules, pollen maturing, and style enlarging. Be = beak; Ov = ovary. (**G**) One mature floret, *stage 13*, with stigma lobes open and receptive. Pa = pappus; Pe = petals, lower 1/3rd tubular, upper 2/3rd ligulate; An = anthers arranged in a ring; St = style and stigmas; insert: mature fruit (*Cypsela*), *stage 16*, with a long beak and opened pappus. © Kitty Vijverberg (**A**,**E**,**G**, insert), Julia Mars (**B**–**D**,**F**), Niki Vorgia (**G**).

*Taraxacum officinale* (F.H. Wigg.) *s.l*. is a perennial herb of the subfamily Cichorioideae, characterized by many (120–200) ligulate, homomorphic, hermaphroditic florets on a radially symmetrical capitulum (Figure 1A) [54,55]. The species is widespread in the temperate zones of Europe and South East Asia and exotic in North America and parts of the other continents. Dandelions start flowering in spring, and usually produce up to ~7 flower heads, each on a hollow, leafless stem, whereas they flower at lower frequencies year-round. The genus is a complex taxonomic group, since it includes sexually (needs pollination) and asexually (apomictic; produces clonal seeds in the absence of fertilization) reproducing genotypes [56,57]. Depending on the definition, up to c. 60 sections (morphological, non-hybridizing units) with over 2000 (micro-)species are recognized [58]. Despite this, the design of the florets is largely conserved, with differences notably in aspects of the petal colour (white, yellow, orange, and, e.g., dark tips or abaxially) [59] and coiling of the ligules. Each floret has numerous pappus parts in a whorl at the base of the corolla. The pappus is placed on a shorter or longer stalk named a *beak* (also *rostrum*; up to ~1 cm long) and opens into a parachute or disk-like structure at maturity. Aerodynamic studies confirmed that the pappus is a highly effective flight mechanism that generates a *lift force* needed to balance fruit weight and an axis-symmetric, recirculating *vortex ring* to stabilize when in cruise conditions [1,4]. To allow adequate permeability for the flow to create a separate vortex ring, the number of pappus parts is important and optimal at ~100 parts, equalling the number commonly found in dandelions. This suggests that the number of pappus parts plays an important role in dispersal capacity. Dispersal potential of *T. officinale* fruits was found to be 1–3 m on average, to be positively correlated to pappus part length, and to be negatively correlated to fruit weight [2,3].

### 1.5. Research Question and Aim

Our main question is whether the pappus in Asteraceae is homologous to the sepals, which we investigated using the common dandelion as a model. We also aim to develop reference data of use in development and evolution studies with a focus on changes in dispersal strategies in response to changing environments. To achieve these goals, we investigated floral organ development in dandelions morphologically and molecularly, with a special emphasis on the pappus. We investigated young dandelion florets with various micromorphological methods; defined the different stages of floral organ initiation, maturation, and seed formation in dandelion; and developed the use of qPCR for dandelion floral tissues by identifying and analysing homologous A- (*AP*1) and B- (*PI*) genes of floral development and verifying reference genes. 

## 2. Results

### 2.1. Floral Organ Ontogeny and Vasculature

To unravel pappus development, we analysed *T. officinale* buds from their initiation up to anthesis with light microscopy, histology, scanning electron microscopy (SEM), and micro-computerized tomography (μCT) scanning (see Materials and Methods, Section 4.1, Section 4.2, Section 4.3, Section 4.4). Buds from plants originating from different locations in Europe, including plants with different genotypes that were grown in a greenhouse and from outside, were analysed; 2–3 buds of at least two different plants per stage per method were studied (see Section 4.1). Based on the results, we defined stages of floral organ initiation (Table 1; *stages 1–6*), floral maturation (*stages 7–12*), and seed formation (*stages 13–16*) in dandelion, all well-confirmed by all buds analysed. Each of the methods highlighted different aspects of the floral organ initiation, development, and vasculature and/or enhanced each other in our conclusions. Measurements resolved the stages in which growth was relatively fast: in the upper floral part during *stages 9–12* and in the lower part, which includes the beak and ovary, during *stages 12–15* (indicated in green in Table 1). 

**Figure 2:**
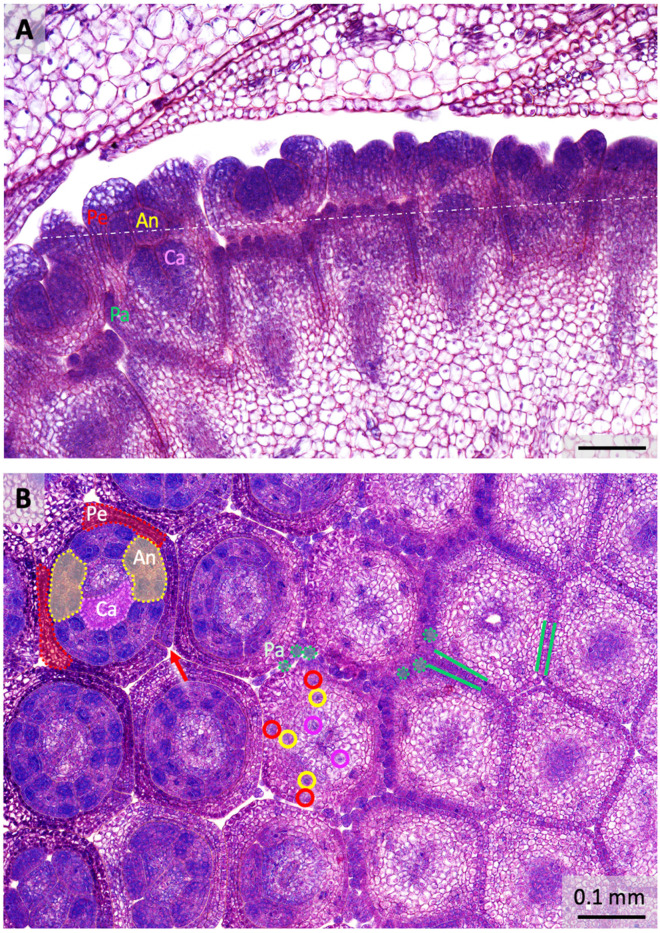
Histology of floral organogenesis in dandelion capitula. (**A**) Vertical section with six florets (*stage 4;* Table 1), showing that pappus parts form a ring at the base of the corolla. Petals do not yet cover the top, anthers are densely celled, and carpels are developing. Pa (green) = pappus; Pe (red) = petals; An (yellow) = anthers (Stamen); Ca (pink) = carpels (Pistil); dashed line = cutting edge of the cross-section in B. (**B**) Cross-section with three rows of five florets (*stage 5*) gradually descending through the flower to ovary from left to right. Coloured areas indicate some of the floral organs and coloured circles some of the vascular bundles; green lines visualize the initiation of the pappus via a transition from one to two cells in the outer cell layer; green dotted circles denote some of the pappus parts higher-up in the floret; pink circles indicate the bundles to the two carpels; red and yellow circles denote three of the five bifurcations to a petal and an anther, while the other two are yet unsplit (right in the floret); red arrow points to the unfused zone between two petals to form the ligule. Stain: Methylene blue/azure II/basic fuchsin. © Marjan Kraaij.

Direct and light microscopical analysis showed that floral primordia in dandelion are already pronounced in about the youngest harvestable stage of buds (defined as *stage 2*; Table 1; Figure 1B). Involucral bracts are then massively present and well-surrounding and -covering the inflorescence receptacle with the initiating florets. Histological analysis showed that slightly later, the floral organs are clearly defined and visible as separate entities (defined as *stage 4*; Figure 2A). The subsequent stages are characterized by elongation of the upper floral part (above the beak), including the pappus, corolla, stamens with anthers, and style with stigmas (defined as *stages 7–13;* Figure 1C–G). Pappus particularly elongates rapidly relative to the petals in *stages 9–12* (Table 1). In these stages, the pigmentation of the petals also takes place, while the corollas form a tube and cover the top of the florets. At anthesis (*stage 12*; Figure 1F), the involucral bracts open and the upper two thirds of the corolla unfolds into a ligule. The style then quickly elongates and collects the mature pollen at its outside while passing the anther tube, presenting it for cross-pollination. Shortly thereafter, the stigmas open and become receptive for cross-pollination themselves (*stage 13*; Figure 1G). After fertilization, the ovary and beak start to elongate quickly, and the upper floral part degenerates except for the pappus (defined as *stages 14–15*). The beak enlarges up to ten times, while the pappus remains the same length and opens like a *parachute* (*stage 16*; Figure 1G insert). At day 12 after pollination (DAP), the dispersal unit (pappus plus fruit) achieves maturity and is ready to break off for wind dispersal. 

Histological analysis showed that the floral organs in dandelions are clearly visible in florets of 0.2 mm (*stage 4*; Table 1; Figure 2A). In a vertical section, it can be seen that the pappus is located in a ring at the base of the corolla and the pappus parts have already developed. The petals do not yet cover the top of the floret, the anthers are densely celled, and the two carpels have started to form the ovary (Figure 2A). The petals initiate first, from five corolla lobes (also petal primordia; *stage 2*), on the plug-like floret primordia, and are ahead in growth. The stamens and pappus initiate second, virtually simultane-ously (*stage 3*), and the carpels initiate shortly thereafter (*stage 4*). In a cross-section (*stage 5*; Figure 2B), the initiation of the pappus can be clearly followed: first there is a transition of the outer cell layer from one to two cells (depicted between green lines), then, slightly higher up in the floret, there is a transition to individual pappus parts of three cells in diameter (some depicted in green dotted circles). At the height where the pappus initiates, two of the five main bundles split to the carpels (encircled in pink). Higher-up in the floret, all five main bundles bifurcate to supply a petal and an anther (encircled in red and yellow, respectively). The cross-section also showed that the petals (two indicated in red in the upper left floret) and anthers (two in yellow) alternate to each other. In contrast, the vascular bundles of the petals and anthers do not alternate, but are opposite, meaning that the lateral petal bundles have evolved into the main bundles, while the median petal bundles remain undetected. The lateral petal bundles of two adjacent petals are fused into one bundle, probably contributing to their pronouncedness, and located in the fusion zones between adjacent petals. In the tubular part of the corolla, a total of five fusion zones between petals are found, whereas in the ligulate part one of these zones lacks fusion (red arrow). Vascular bundles to the pappus were not detected.

**Figure 3:**
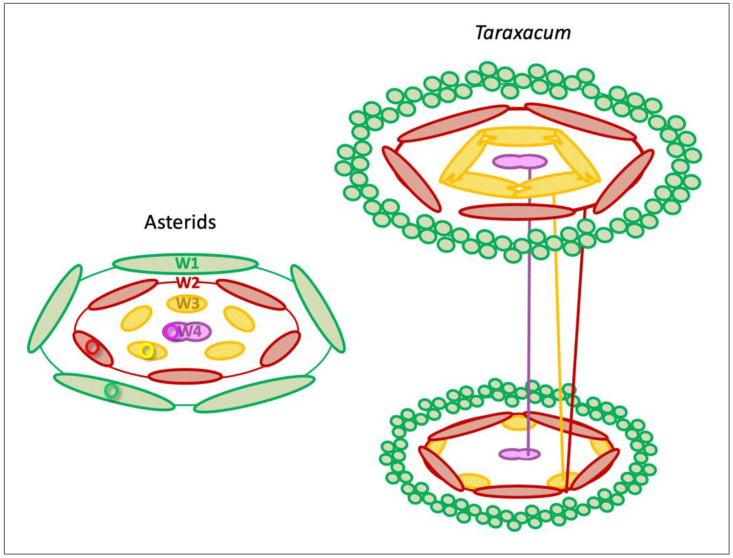
Floral diagram of the Asteridae versus dandelion. (**Left**) The floral diagram representative of the Asteridae, modified from [25], showing the four floral whorls (W1–W4) and pentamerous bauplan, with five sepals (green), five petals (red), five stamens (yellow), and two carpels (pink), with one of each vascular bundle indicated (coloured circles). (**Right**) Floral diagrams representative of the upper and lower part of the dandelion floret, showing deviations from the common diagram in three respects: (1) in the outer floral whorl, the five sepals are absent, instead numerous pappus parts are found; (2) a central or major bundle to the pappus is lacking; (3) the median bundles to the petals are undetected, whereas major lateral bundles are present in the fusion zones between adjacent petals instead; one of each vascular bundle indicated (coloured lines). In the lower part of the dandelion floret, the petals are fused to a tube and the stamen filaments associate with the fusion zones. In the upper part of the floret, one of the fusion zones between petals is lifted, where the corolla forms a ligule and anthers form a continuous ring (see Figure 1G).

The results are summarized in the basic floral diagram representative for the majority of Asteridae (Figure 3) [25], highlighting the deviations found in dandelion. The first is the absence of sepals in whorl 1, where numerous pappus parts are found instead. Secondly, we confirm the assumption that a central or major bundle to the pappus is absent. Thirdly, major petal bundles are located at the fusion zones between petals rather than in a median position. Lower in the florets, the thin stamen filaments are fused to the petals, whereas higher in the floret, the anthers are arranged in a continuous ring.

**Figure 4:**
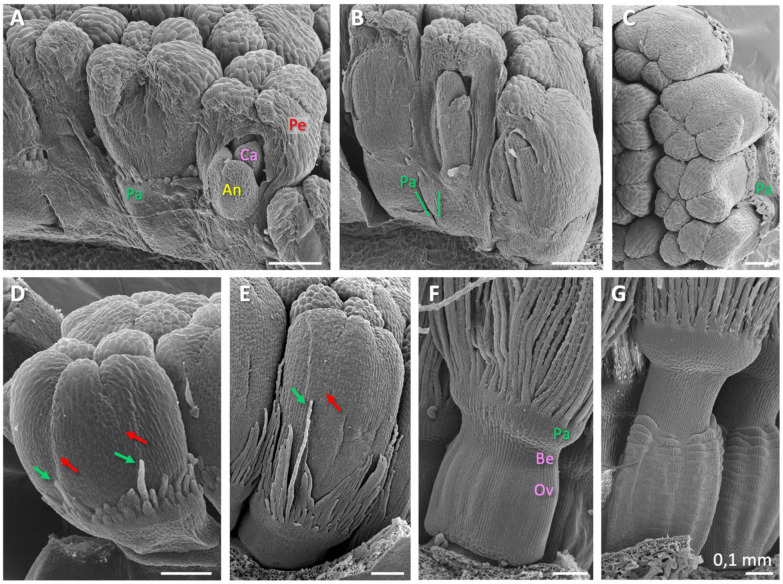
Scanning electron microscopy analysis of dandelion florets at young developmental stages. (**A**) Part of a capitulum of *stage 5* (Table 1), with all floral organs formed, the petals covering the top of the florets, and the carpels lower than the anthers. Pa (green) = pappus; Pe (red) = petals; An (yellow) = anthers (stamen); Ca (pink) = carpels (pistil). (**B**) Some florets of *stage 6*, showing organ elongation, and the carpels past the anthers. Green lines indicate part of the pappus ring initiation zone at the base of the petals, with a recently initiated pappus part on top. (**C**) The same florets of B viewed from the top, showing 2–3 rows of pappus parts separated from the corolla. (**D**) Floret of *stage 5*, showing that pappus parts (green arrows) located at the petal fusion zones (red arrows), the alternipetal position where the sepals are expected (Figure 3), are ahead in growth. (**E**) Floret of *stage 7*, confirming the growth advance of pappus parts located at the petal fusion zones, and showing relatively increased growth of the pappus parts; the ovary and beak start to pronounce. (**F**) Floret of *stage 8*, with mature pappus parts on which scales have started to form, a pronounced pappus ring, and a clearly formed beak (Be) and ovary (Ov), with vertical lines on the fruit. (**G**) Fruit/Floret of *stage 10*, showing the elongated beak and ovary and horizontal lines on fruit. © Bertie Joan van Heuven.

SEM analysis largely contributed to the definition of the floral stages in dandelion (Table 1) and beautifully gave a 3D impression of the florets at young developmental stages (Figure 4). The results showed the different organs shortly after their initiation (*stages 5-6*; Figure 4A–D). They visualized how the pappus initiates as a ring primordium (Figure 4B green lines, compare with Figure 2B), on top of which the small individual pappus parts develop in two or three rows (Figure 4D). The ring primordium of the pappus can be interpreted as the result of the *congenital* fusion of the sepal primordia in the first whorl. Interestingly, pappus parts at the petal fusion zones, which is in the alternipetalous position where regular sepals would be expected (Figure 3), are usually ahead in growth (Figure 4D,E), which may imply sepal identity. The growth advance of pappus parts at petal fusion zones was confirmed by μCT scanning analysis (Supplementary Figure S1A). Evidence for the initiation of five pappus lobes prior to the formation of the pappus ring was not confirmed. Indications for a *pentamerous* origin of the pappus, thus for the presence of five growth units from which the pappus develops rather than from a continuous ring, were very weak and incidental (Supplementary Figure S1B). At *stage 7*, the pappus parts start to elongate relative to the petals (Figure 4E, also Figure 1C,D). The ovary and beak are unpronounced in young florets, but this changes in *stage 7*, and they are clearly defined in *stage 8* (Ov, Be; Figure 4F). The pappus ring has developed into an obvious ring at this stage (Pa; Figure 4F), but it is not entirely clear where and how it demarcates in relation to the *floret receptacle* (c. q., flower bottom). Vertical sections of *stage 8* florets shed some more light on this by clearly showing the transition from one to two cell layers at the base of the pappus ring (Supplementary Figure S2, green arrow) and a similar cell type and staining pattern of the cells in this ring, supporting that it belongs to the pappus and represents whorl 1. These sections also indicated that the ovary consists of carpel tissue (whorl 4), while surrounded by a layer of another cell type. This was also prominent in the younger florets (Figure 2A). We interpret this layer as the floret receptacle (indicated with a white dotted line in Supplementary Figure S2; discussed in Section 3.3). The beak is still short in *stage 8*, but also consists of carpellary tissue, with another cell type at the outside and top, probably representing the floret receptacle and at the top some cells of whorl 1 (white arrow in Supplementary Figure S2). In sections of older florets (*stages 10–12*; not shown), such outgrowth of other cells along the outside of the elongating beak was also seen, but its nature was not unambiguously established. Overall, our data suggested the position of the floret receptacle in dandelion and its delineation to the pappus ring, and defined the composition of the beak as primarily consisting of carpel tissue and being entirely or largely independent of the pappus. At *stage 10*, the ovary and beak further elongate (Figure 4G), however, their true elongation occurs after pollination and the onset of embryo formation (*stages 12–16*).

**Figure 5:**
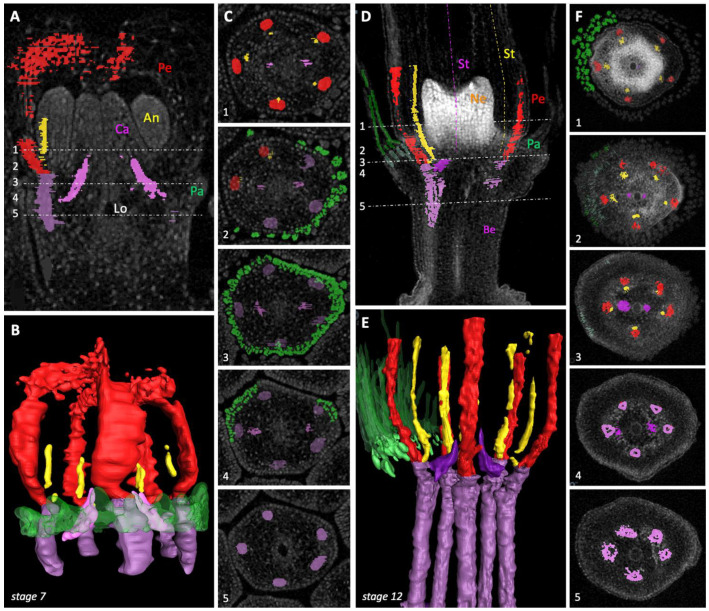
Micro-computed tomography scanning analysis of the floral vasculature and pappus development in dandelion. Young floret (*stage 7*; Table 1; (**A**–**C**)) and floret at anthesis (*stage 12*; (**D**–**F**)) presented in a vertical section (**A**,**D**), 3D-model ((**B**,**E**); Supplementary Videos SV1, SV2), and five horizontal sections (**C**,**F**) of which the cutting edges are indicated by numbers and white dashed lines in the vertical sections. Pa (green) = pappus; Pe (red) = petal bundle; An/St (yellow) = anther/stamen bundle; Ca/St (pink 1) = carpel/style bundle; (pink 2) = main bundle; Lo = locule; Be = beak; Ne = nectaries. (**A**) Segmentation of one of the five main bundles, its split to one of the carpels and bifurcation to a petal and an anther, and the bundle to the second carpel. (**B**) 3D-model showing the entire vasculature of the floret, and the pappus. Main bundles of the pappus are absent. The movie (Supplementary Videos SV1) also visualizes the bifurcation of one of the petal bundles. (**C**) Exact position of vascular bundles, their splits, and context to the developing pappus, confirming that the pappus initiates at the height where two main bundles split to the carpels (Number 4). (**D**) In mature florets, capillaries (light green) to the pappus parts (dark green) are present in the upper part of the pappus ring, and the split of bundles to the carpels and petal/anthers occur in a more compressed region (Numbers 3 and 4). Additionally, nectaries (abundant white cells) and the beak are clearly visible. (**E**) 3D-model showing the capillaries (solid green) to the pappus parts (green-transparent). Main bundles are much enlarged as compared to the young floret and bundles or capillaries to the nectaries are absent. The nectaries are also shown in the movie (Supplementary Videos SV2). (**F**) The sections show that the capillaries to the pappus parts become visible at the height where the main bundles split to the petals and anthers (Number 3). A high-resolution scan showed that they actually branch off earlier, at the bifurcation of the carpels (Supplementary Figure S3). © Monique Welten.

The μCT scanning analysis revealed the branching pattern of the vascular bundles to the floral organs in dandelion (Figure 5). It visualized the split of two of the five main bundles to supply the two carpels and, slightly higher up in the floret, the bifurcation of all five main bundles to supply a petal and a stamen. The branching pattern supported the identity of the different floral organs as described above and their position with respect to each other. In young florets (*stage 7*; Figure 5A–C; Supplementary Video SV1), the region in which the branching to the carpels and petals and anthers occurs is more stretched relative to older florets (*stage 12*; Figure 5D–F; Supplementary Video SV2). In the young floret, it was confirmed that the initiation of the pappus occurs at the height where the two bundles split to the carpels (Number 4, Figure 5C) via the transition from one to two outer cell layers and subsequent development of individual pappus parts (Numbers 2 and 3). The absence of main vascular bundles to the pappus was supported. Additionally, the opposite arrangement of the vascular bundles to the petals (red) and anthers (yellow) was confirmed. A movie visualized one of the five petal bundles splitting into the two lateral bundles, where the corolla forms a ligule (Supplementary Video SV1). In florets at anthesis (*stage 12*; Figure 5D–F; Supplementary Video SV2), it was found that the pappus parts arise at the top of the pappus ring and capillaries to the pappus parts veined the upper part of it. Since the branching region is somewhat compressed in older florets, all splits concentrate in Numbers 3 and 4 (Figure 5F), while the capillaries to the pappus parts became visible at first in Number 3. In high-resolution scanning, it became apparent that the capillaries to the pappus parts actually branch-off lower in the floret, just around the split of two main bundles to the carpels (Supplementary Figure S3). The floral nectaries initiate relatively late during development (*stage 7*). They are clearly visible in *stage 8* (Supplementary Figure S2) and are massively present and densely celled in this and later stages (e.g., *stage 12*, Figure 5D). The nectaries lack main vascular bundles, whereas the presence of capillary bundles remained unresolved due to the high cell density. The nectaries, however, were found to form a ring at their base that connects to the vascular bundles of both the anthers and petals, presumably providing their supply in this way (Supplementary Figure S4). 

**Figure 6:**
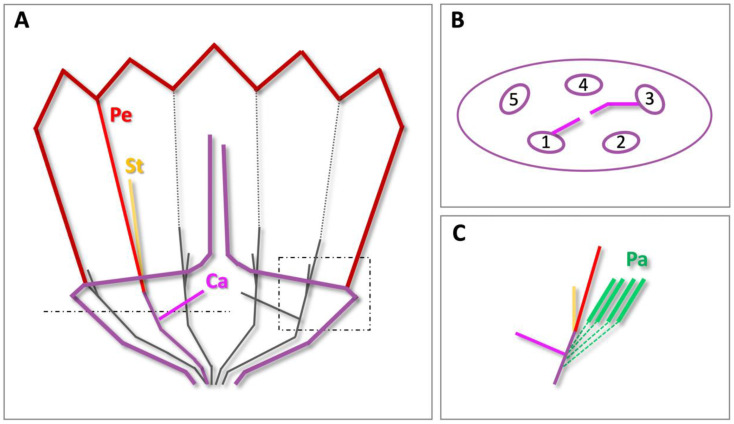
Summary of the vasculature in dandelion florets. The diagrams are representative of species of the Asteraceae, modified from [60]. (**A**) A longitudinal diagram showing the two splits to the carpels (Ca) and five splits to the petals (Pe) and stamens (St), with one of each bundle indicated in colour: Petal bundle (red); stamen bundle (yellow); main bundle (dark pink); carpel bundle (bright pink). Main bundles to the pappus are absent in dandelions, but capillaries to pappus parts are present in mature dandelion florets (see Figure 5D,E; Supplementary Figure S3 and Video SV2). Outer lines corolla (dark red); outer lines ovary and style (dark pink); broken line = cutting edge of the transverse diagram in (**B**); broken line box = enlargement in (**C**). (**B**) A transverse diagram showing the ovarian vasculature, with numbers denoting the five main bundles that each bifurcate to a petal and a stamen, and solid lines indicating the two bundles to the carpels, with one moved to the more central position between bundles 3 and 4 (see also Figure 5C; Supplementary Figure S5). (**C**) Capillary bundles (dashed green lines) to the pappus parts (Pa; solid green lines) and their branching from the main bundles just around where they split to the carpels.

The results of the vasculature are summarized in floral diagrams representative of species of the Asteraceae (Figure 6) [60]. In the longitudinal diagram, the common vasculature is confirmed for dandelion florets (Figure 6A). In the transverse diagram, one of the common ovarian vasculatures was confirmed for dandelion, with encircled numbers indicating the main bundles that each split to supply a petal and an anther, and the solid lines indicating the two bundles that supply the carpels, the latter including the movement of one of the two bundles to a more central position (Figure 6B). Additional visualization of this ovarian vasculature in a young (*stage 7*) and older (*stage 12*) floret of dandelion is shown in Supplementary Figure S5. The absence of main bundles to the pappus was confirmed, instead, capillaries to supply the pappus parts were found in mature florets. These branch-off from the main bundles just above and around the split to the carpels (Figure 6C), at the height where the base of the pappus ring is located.

### 2.2. Floral Organ Gene Expression

To determine the homology of the pappus to the sepals, we analysed the expression of *T. officinale* homologous A- and B-genes of floral development: *Tof-APETALA*1 (*Tof-AP*1) and *Tof-PISTILLATA* (*Tof-PI*), in different floral tissues and stages (see Materials and Methods Section 4.5, Section 4.6, Section 4.7). The analysis also included the identification and verification of two reference genes in dandelion, *Tof-UBIQUITIN CONJUGATING ENZYME*9 (*Tof-UBC*9) and *Tof-PROTEIN PHOSPHATASE*2A (*Tof-PP*2A), chosen on the basis of good performance in lettuce [61]. All four genes were defined based on sequence similarities with published sequences of Arabidopsis and lettuce to those of *Taraxacum kok-saghyz* [62]. Primers were designed in the conserved regions of these genes and verified experimentally for their use in *T. officinale* (see Section 4.5).

**Figure 7:**
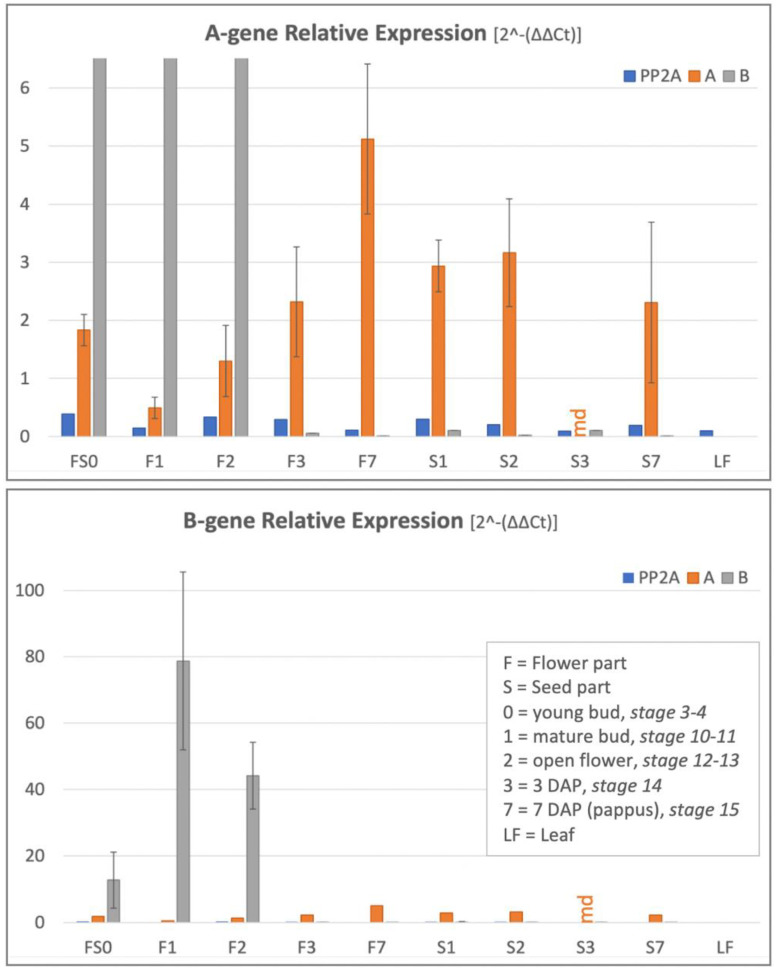
Relative A-gene (*Tof-APETALA*1) and B-gene (*Tof-PISTILLATA*) expression in dandelion floral tissues at different developmental stages. F = upper floral tissues (including the pappus, petals, stamen with anthers, style with stigmas, and upper half of the beak); S = lower floral tissues (including the ovaries and lower half of the beak); LF = leaf; numbers are explained in the graph and stages in Table 1; DAP = days after pollination; F7 = pappus only; md = missing data. The two graphs are the same, but differently scaled to highlight the small but relevant variation in the A-gene expression ((**upper**) graph) and ~20× higher expression of the B-gene compared to the A-gene ((**lower**) graph). *Tof-AP*1 (orange bars) is weakly expressed in all tissues and stages analysed with a gradual increase in maturing florets (F1 to F3), being highest in the mature pappus (F7). *Tof-PI* (grey bars) is highly expressed in the floral tissues that contain petals and anthers up to anthesis (F0 to F2) and absent in pappus (F7). *Tof-PP*2A (blue bars) is a second reference gene, showing the same pattern as *Tof-UBC*9. These results verify the ABC-model of floral development, supporting the homology of the pappus with the sepals.

We analysed the expression of *Tof-AP*1 and *Tof-PI* using either *Tof-UBC*9 or *Tof-PP*2A as a reference. Both genes showed a similar expression pattern, both in height and variation between the floral tissues, and thus are both valid reference genes and equally usable. We used *Tof-UBC*9 in the final analysis (Figure 7), while we illustrate the similar performance of the two reference genes by regarding *Tof-PP*2A as a sample (blue bars in Figure 7), showing that its relative expression compared to *Tof-UBC*9 is close to zero in all tissues. Having stable reference genes over different floral tissues is very useful for future qPCR analysis in dandelions, and since their good performance in lettuce, putatively also in a wider range of Asteraceae.

Our expression analysis was performed on either the upper part of the floret, named F, or the lower floral part, named S, separated by cutting through the beak (see Section 4.6). The upper floral fraction thus includes the pappus, petal, stamens, style and stigmas and upper half of the beak, while the lower floral part includes the ovary, lower half of the beak, and some remains of the inflorescence receptacle. Results showed *Tof-AP*1 expressed in the *upper* floral fractions, increasing with maturity from closed buds (F1) to open flowers (F2), old flowers 3 DAP (F3), and being highest in the pappus at 7 DAP (F7). In addition, *Tof-AP*1 was weak expressed in all *lower* floral fractions, which we interpret as a result of floral meristematic and/or vascular tissues in these fractions (see Section 1.3). *Tof-PI* was highly expressed in the upper floral tissues only, up to anthesis (F0–F2), whereas it was completely absent from the old floral tissue (F3), pappus (F7), and lower floral fractions at all stages (S0–S7).

## 3. Discussion

### 3.1. Floral Organogenesis and Stage Definition

To decide on pappus–sepal homology, we first observed the floral development of dandelion micromorphologically (Figure 1, Figure 2, Figure 3, Figure 4, Figure 5 and Figure 6; Supplementary Figures S1–S5 and Video’s SV1 and SV2) and defined the different stages of floral initiation, floral maturation, and seed formation (Table 1). Well-defined stages of floral ontogenesis are available for some model species, particularly Arabidopsis [63], but in the Asteraceae the main focus has been on the inflorescence and not florets development, for instance in Gerbera [64], with additional floral initiation stages in [65], and recent studies in Chrysanthemum [66], lettuce [53], and the rubber dandelion (*T. kok-saghyz*) [67]. Our study concerns the floret development only, starting with the floral primordium as *stage 1*, and is aimed as a reference for dandelions and, where possible, other Asteraceae. Our definitions of floral organ initiation largely correspond with and are tuned to those reported for Gerbera [65].

We demonstrate that the initiation of the corolla (*stage 2*; Table 1) is first, stamens is second (*stage 3*), and carpels is third (*stage 4*) in dandelions. The pappus ring becomes visible at the outside of the floret at the moment the stamens can be seen (*stage 3*). This is *heterochronic* compared to the origin of the sepals (whorl 1) in most other angiosperms, in which they initiate first and the petals (whorl 2) second, e.g., [63], while it is homologous with respect to the *position* of the sepals (whorl 1) in other angiosperms. According to the histological sections (Figure 2A), the carpels initiate slightly earlier than they become visible externally, being embedded within the tissue. The initiation of the pappus, stamens, and carpels thus occurs in rapid succession. Similar results were found by Harris [68] and Sattler [29]. Harris [68] provided detailed SEM-observations of inflorescence and floret development in a range of Asteraceae, including five species from the Lactuceae that are closely related to dandelions. For three of these species, the stamens originated second and the pappus third, whereas for the other two the opposite was found. According to Harris, the order of initiation of whorls in Asteraceae florets is always corolla first, then stamens, then gynoecium, while the initiation of the pappus is plastic with regard to its timing, but seldom first. Heterochronic initiation of the corolla relative to the pappus in the Asteraceae was also reported by Small [28]. Harris [68] also mentioned that the order of organ initiation is constant within one species, and that organs of one type usually initiate simultaneously. We confirmed this pattern in dandelions but found that the initiation of the individual pappus parts, thereafter, is not always synchronous within and between dandelions. Harris’ [68] study lacked histological analysis, but the presence of carpels before their visibility from the outside is likely valid also for other Asteraceae. Apart from the heterochronic initiation of the pappus, the time and place of organ initiation in dandelion florets and Asteraceae in general corresponds to the pattern in most other angiosperms, confirming their shared ancestral building plan. 

After the dandelion floral organs are initiated (*stage 6*), they mature, particularly by elongation in the upper floral part (Table 1, green). In addition, the floral nectaries initiate, petals pigmentate, and ovary and beak delineate (Figure 1C–G and Figure 4E–G). Our observations indicated that the ring below the pappus parts entirely belongs to the pappus and is of whorl 1 nature (Figure 4F,G; Supplementary Figure S2; Section 2.1). The ovary and beak represent whorl 4, while on the outside they contain a different cell type, which we interpret as derived from the floret receptacle (Supplementary Figure S2; Section 2.1). At the end of floret maturation, the corolla unfolds, the style grows through the anther tube (*stage 12*), and the stigmas become receptive to cross-pollination (*stage 13*). This pattern of maturation is also common in other Asteraceae, e.g., in lettuce [53] and Gerbera [64], as well as outside the Asteraceae, e.g., in Arabidopsis [63]. After pollination (or spontaneously in the case of apomictic dandelions [56,57]), the fruiting and seed maturation begins, causing rapid elongation of the lower floral part: the ovary and beak, and degeneration of the upper floral part: the petals, stamens, style, and stigmas, but *not* the pappus. The beak lengthens ~10x, to ~1 cm, in dandelions during this period, while the length of the pappus remains virtually the same. Unlike the usual degeneration of the sepals, the pappus remains intact, apparently to fulfil its new function in seed dispersal. At the end of this phase (*stage 16*), the cypselae are brownish and ready to break off (Figure 1G insert).

### 3.2. Sepaloid Origin of the Pappus

A strong indication of a sepaloid origin of the pappus would be the finding of five pappus primordia alternating with the petals, being an indication of the *pentamerous* bauplan of the florets (Figure 3). Following the *phyllome* theory, which assumes that the pappus derives from a *leaf* structure, a sepal [27,28], it is suggested that the ancestral Asteraceae contain a five-lobed pappus and this either has evolved in numerous pappus parts in some more derived lineages of the Asteraceae or the absence of pappus parts in some other lineages [29,69]. Clear evidence for the development of the pappus from five primordia was not observed in dandelions. However, stronger growth of the pappus parts opposite to the fusion zones of the petals, which indicates an alternipetalous positioning of the growth zones, was evident (Figure 4D,E; Supplementary Figure S1A), and some evidence was found for the pappus growth in five units (Supplementary Figure S1B). We also showed that the pappus ring that precedes the formation of individual pappus parts was formed via the transition of cells in the outer cell layer from one to two cells (Figure 2A), potentially indicating the congenital fusion of the sepals and supporting the whorl 1 nature of the pappus. In addition, the ring that later forms during development (Figure 4F) consists of a similar cell type (Supplementary Figure S2), supporting that it belongs entirely to the pappus. Together, these results support a sepaloid ancestry of the highly derived pappus in dandelion. Harris [68] reported the formation of five pappus lobes in one of the five Lactuceae species studied, *Malacothrix saxatilis*. These were in the alternipetalous position, but this species forms two types of pappus, with the five lobes probably representing the outer row and not the inner one that consists of many pappus parts, more similar to these in dandelions. In more mature florets of *M. saxatilis*, five pappus growth units could be recognized, with the longest pappus parts at the fusion zones of the petals (see Figure 87 plate 12 in [68]) as in dandelion. Another species, *Eutrochium fistulosum* (Heliantheae alliance; Asteroideae), showed five lobes in combination with numerous pappus parts, and a third, also from the Heliantheae alliance, *Gaillardia aestivalis*, showed individual pappus lobes to produce 6–10 scale-like pappus parts. In *Tragopogon portensis* (Cichorioideae), another species with a high number of pappus parts, the inception of the pappus was found to begin also with five primordia [29]. The majority of species studied by Harris [68], however, showed a pappus ring preceding the origin of many pappus part primordia on this ring, as we found in *T. officinale*. In *T. kok-saghyz*, pappus part primordia of similar sizes were seen at both the alternipetalous and antipetalous position (Figure 3F in [67]). 

Alternative explanations might be possible, but are less supported by our data. One other explanation refers to *physical constraints* [70], on the basis of which one may argue that the pappus parts at the alternipetalous positions have more space to develop. Another explanation is that the development of the ring primordium *arrests* and the parts that develop from this ring have another nature, e.g., are trichomes or scales, following the *trichome* theory [27,30]. If the ring is a modified calyx and the pappus parts are of another nature, the sepaloid origin of the pappus is, however, still supported, while the pappus parts are more derived structures. It would imply a breakdown of the sepal developmental program and build-up of the trichome formation program, which is quite advanced, e.g., [71]. We also found scales on the pappus parts (Figure 4F,G), which is unusual on both trichomes and scales. Our morphological data, therefore, lack strong support for the alternative hypotheses, while our molecular data does not support them either (see next paragraph). 

Further support for a sepaloid origin of the pappus would be floral A-gene expression in the tissues that include the pappus and in the pappus alone, and absence of floral B-gene expression in the pappus [31,34]. Analysis of dandelion floral tissues indeed confirms this pattern (Figure 7), by showing low *Tof-AP*1 expression in all floral tissues, with the highest expression in mature pappus alone, and high *Tof-PI* expression confined to the tissues that include the petals and the stamens, being absent in the pappus. More refined expression studies, including the analysis of individual organs and performing in situ hybridizations, are needed to finally resolve which tissues could be considered whorl 1. This first needs a further characterization of the underlying genes in *T. officinale*. In the closely related species lettuce, floral developmental genes were identified, including *Ls-AP*1 (*LsMADS*55) and *Ls-PI* (*LsMADS*03), and their expression in young florets reported [53]. Sequence homology of these genes to published sequences from Arabidopsis supports their floral gene specificity, while their sequence similarity to sequences from *T. kok-saghyz* [62], and the absence of alternative family members therein, support their homology also in *T. officinale*. Results in lettuce showed that *LsMADS*55 is expressed in the pappus and, somewhat weaker, in the tissue that surrounds the ovary. The former supports the sepaloid origin of the pappus, making it an interesting pappus-specific gene in Asteraceae, although lacking complete A-functionality, since its expression in the petals was absent. The expression in the ovary wall is discussed below (Section 3.3). *LsMADS*03 showed strong expression in the petals and stamens, similar to our results in dandelion, and confirming B-functionality. The expression results in dandelion as well as lettuce, thus support the homology of the pappus to the sepals, but with various *AP*1-like expression patterns.

It is generally thought that the diversity of flower shapes and functions is the result of the plasticity and differential activity of the flower meristems [72], which produce the different floral organs over time and space. Our results in dandelion illustrate this. They show that pappus–sepal homology is confirmed with respect to the place of the pappus but differs in its timing of initiation. The expression patterns of the underlying floral A- and B-genes support the sepal identity of the pappus, while differences in A-gene functionality are common, both outside and within the Asteraceae (Section 1.3, Section 3.3). Possibly, whorl 1 meristems and the underlying expression of *AP*1-like genes, are relatively sensitive to change, perhaps because whorl 1 has a less direct role in reproduction compared to the other whorls. If so, whorl 1 may provide an important possibility for flowers to adapt and achieve a new function. In dandelions, and the Asteraceae more generally, these adaptations particularly shifted towards a role in dispersal. 

### 3.3. Development of the Beak and its Connection to the Pappus 

Despite being inseparable parts of dandelion’s seed dispersal-promoting structure, the *beak* and pappus have different origins. The beak is part of the ovary (whorl 4; Figure 2A; Supplementary Figure S2), while the pappus is a modified calyx that initiates on the outside of the florets (whorl 1; Figure 2B and Figure 4B,F; Supplementary Figure S2). Our morphological data showed that the wall of the ovary consists of a different cell type, which probably also continues along the wall of the beak (Supplementary Figure S2), while at the upper outside part of the beak there might be some whorl 1 tissues in addition. To better understand the composition of the beak and the ovary as a whole, it is important to understand the origin of the inferior ovary and location of the floret receptacle. There are two main theories [73,74]. The *appendicular* theory assumes extensive fusion of the lower portions of the floral whorls to each other and the ovary wall during evolution. This implies it is hard to distinguish whorls in this area. The *receptacular* theory suggests that the ovary is partially enclosed by the receptacle tissue. Our micro-morphological data in dandelion support the latter, showing that the carpel/ovary is surrounded by a layer of another cell type that we interpret as a floret receptacle (FR, white dotted line in Supplementary Figure S2; Figure 2A; Section 2.1). Our histological data also showed some evidence for possible fusion of lower parts of floral whorls in the region where the petals and stamen initiate (thin white broken line in Supplementary Figure S2), and along the upper part of the beak/ovary (white arrow). Further identification of homologous ABC(D)E-genes in dandelion and other Asteraceae, and verification of their whorl specificity, will allow more detailed investigations of the whorl composition in this region, e.g., via in situ hybridizations. 

Our hypothesized location of the floret receptacle in dandelions is supported by expression data in lettuce [53] (see also Section 3.2, third paragraph). The A- and C-homologous genes in this species, *LsMADS*55 and *LsMADS*69, showed a mutually exclusive expression pattern in young lettuce florets (~*stages 2–8*; Figure 3 in [53]). *LsMADS*55 was expressed in the pappus and ovary wall, with the latter corresponding to the region that we define as the floret receptacle in dandelion (FR, *outside* of the white dotted line in Supplementary Figure S2). *LsMADS*69 was expressed in the ovary, style and stigmas, and stamen/anthers, with the former corresponding to the region *inside* the white dotted line in dandelion (Supplementary Figure S2). The three floral B-genes in lettuce all showed expression in the petals and stamens only, from above the thin white broken line in dandelion (Supplementary Figure S2). The A-homologous gene *GSQUA*1 in Gerbera was also expressed in the ovary wall, similar to *LsMADS*55, and additionally in the connecting parts in the inflorescence receptacle (Figure 3 in [46]). In fact, the floret receptacle is a small, protruding part of the inflorescence receptacle. It was suggested, on the basis of its position that *GSQUA*1 was expressed in the developing vascular bundles. This is consistent with what we see in the histological sections of dandelion, where there are vascular bundles in the ovary wall/floret receptacle (dark stained cells near the white arrow in Supplementary Figure S2). Our μCT scanning analyses also clearly showed the vascular bundles in the ovary wall, also continuing along the beak (Supplementary Figure S4B). Our data thus indicate that the beak wall consists of receptacle derived tissue. Whether these expression patterns also hold for dandelions is unknown, but based on the combination of results it is expected. It is especially interesting that the dispersal-promoting structure of the Asteraceae is a highly derived *composite* organ evolved from the fusion of whorl 1, the pappus, which develops mainly before fertilization, with whorl 4, the beak/upper part of the fruit, which matures mainly after fertilization. 

### 3.4. Floral Vasculature

The vasculature in florets of dandelion confirms the general branching pattern in species of the Asteraceae (Figure 6A) [60]. Main bundles to the pappus are absent in dandelion, as is common in Asteraceae, while present to supply the sepals in most other angiosperms. We found capillaries to the pappus parts in mature dandelion florets, branching off from the main bundles around the split to the carpels (Figure 5D–F; Supplementary Figure S3). Other indications for bundles to or in the pappus have been reported for some species, for instance, in *Microseris bigelovii* (Lactuceae), which has a five-part pappus, and *M. pygmaea*, with a ten-part pappus, a constant number of ten *pro*vascular bundles were found [75]. In some other Asteraceae, including *Porophyllum ruderale* (Heliantheae, Asteroideae) and *Tridax procumbens* (Heliantheae alliance) the presence of veins *in* pappus parts were reported [76]. Thus, the vascular system in Asteraceae shows a number of adaptations depending on the type of pappus and the need for their supply.

Another deviation in dandelions from the typical floral vasculature in angiosperms is the location of the most obvious bundles in the petals. We found that the lateral petal bundles of neighbouring petals had merged into one and were located in the fusion zone between these two petals, while the median petal bundles remained undetected in dandelion florets (Figure 2A, Figure 3 and Figure 5). As a result, petal and anther bundles appear opposite each other, while the organs themselves alternate. Eames [77] described the fusion of lateral veins of the petals in Asteraceae, and Gustafsson [78] reported that the venation at the petal margins is an important character for higher-level systematics of the asterids, with the Asteraceae, Calyceraceae, Goodeniaceae, and Menyanthaceae uniquely sharing this characteristic. In Gerbera, evidence for the presence of major bundles at the petal margins was supported by in situ hybridizations (Figure 3 in [46]). We confirm this characteristic in dandelion and clearly visualize it in 3D-models of a young and mature floret (Figure 5; Supplementary Video’s SV1 and SV2). In addition, we show that one of the five petal bundles bifurcates in the upper two-thirds of the corolla, where the fusion between the petals is absent to form a ligule (Supplementary Video’s SV1).

### 3.5. Evolution of Dispersal Capacity

Our results support the sepal identity of the pappus in dandelion and illustrate that it is fused with the upper part of the fruit, the beak, to form a highly derived dispersal-promoting structure. They demonstrate, together with the different adaptations in the dispersal-promoting structures described for other Asteraceae [24] and variation found in floral A-gene functionality that underlies whorl 1 organ development (Section 1.3, Section 3.2), that the outer floral whorl has been subjected to changes during evolution. The variation in the pappus in the different Asteraceae species, e.g., hairy or scale-like for wind and animal uptake, respectively, together with the enlargement of the beak, has further diversified and improved seed dispersal. The relative susceptibility of whorl 1 to changes may be due to the supposed less direct role in reproduction of this whorl compared to the other whorls, but particularly also to a redundancy of the usual protective function of the sepals in florets taken over by the protection of the whole capitulum by involucral bracts in Asteraceae. The plasticity of whorl 1 makes the organs in this whorl ideal to study for changes in response to rapidly changing environments. Our presented data, stage definitions, and information from the expression analyses provide valuable references for such studies in dandelion and may be useful for studies in other Asteraceae where possible.

## 4. Materials and Methods

### 4.1. Plant Material and Sampling of Buds for Micromorphological Analysis

The dandelions originated from three different populations in mid-Eastern France, obtained by Koen J. F. Verhoeven (F1–3) [79], and three populations in mid- and North Netherlands (N1–3; near Nijmegen, Wageningen and Groningen, respectively). Four of the genotypes were apomictic (F2, N1–3) and two sexual (F1, F3). Between 2–6 plants per population were grown in a greenhouse under 16/8 h light/dark conditions, frost free and a maximum temperature of 20 °C, for a few months up to years. Some buds from the Netherlands were used directly after collection in the field.

Micromorphological analyses were performed on 2–3 buds per stage per method, involving at least two plants that represent the variation among individuals. Additional buds were analysed if further deepening of the results was desired, particularly of the younger stages, e.g., 10 buds for light microscopy, 10 for SEM, five for histology and five for μCT scanning. Buds were harvested and the stems and outer involucral bracts were removed. For direct and light microscopic analysis, fresh buds were used after being cut into halves (Figure 1). For histology, SEM, and μCT scanning analysis, buds were fixed (see Section 4.2, Section 4.3, Section 4.4, respectively), either as a whole or as halves, then transferred to 70% Ethanol (EtOH) and further processed immediately or stored at 4 °C until use.

### 4.2. Histology

Fixation and resin-embedding of *T. officinale* buds of different stages followed Zhang et al. [80] with minor modifications. Buds were fixed in formaldehyde acetic acid (FAA; 3.7% formaldehyde, 5% acetic acid, 50% EtOH) for ~20 h at room temperature under a vacuum obtained by a drawn-up syringe. Dehydration occurred via an EtOH series of 50%, 70%, 90%, 95%, and 2× 100% EtOH in MilliQ water (MQ), each for 1 h and with the application of a vacuum in the same way. Infiltration in Technovit 7100 base liquid (Kulzer, Wehrheim, Germany):EtOH 1:1 was done for 2 h followed by 3:1 for 1 h, 100% T7100 for 1 h, and 100% T7100 overnight, all at room temperature and under vacuum. Before embedding, 0.6 mL Polyethylene Glycol (PEG) 400 per 15 mL T7100 was added to soften the resin block for sectioning [81], after which 1 mL hardener II was added according to the manufacturer’s guidelines. Buds were immediately placed in the right position and, despite polymerization in less than 20 min, also here a vacuum was applied. Samples were sectioned to 5–10 µm thickness using a rotary microtome (Beck, London, UK) with tungsten carbide knives (Sollex AB, Malmö, Sweden).

Tissue staining was performed with either Methylene blue/Azure II/Basic fuchsin [82], resulting in blue nuclei and magenta cell walls (Figure 2), or Etzold (Safranin/Astra blue/Fuchsin) [83], giving red nuclei and blue and magenta cell walls (Supplementary Figure S2).

### 4.3. Scanning Electron Microscopy (SEM)

Buds were fixed in an excess of ice-cold Carnoy (EtOH absolute:Acetic acid glacial 3:1) with the application of a vacuum for 15 min for three times with Carnoy exchanges, and then left overnight in Carnoy in the cold room under weak stirring. The next day, Carnoy was replaced by ice-cold 70–75% EtOH and the samples stored at 4 °C until use. Before scanning, samples were dissected by using a light microscope, while emerging in 70% EtOH, then two times washed in 96% EtOH, each for 20 min., and two times in 96% acetone, each for 30 min. Tissue drying, mounting, and coating followed Pramanik et al. [84]. Samples were observed with a field emission scanning electron microscope (JSM-7600F SEM, JEOL Ltd., Tokyo, Japan) using the settings and picture sizes described [84].

### 4.4. Micro Computed Tomography (μCT) Scanning 

Buds fixed in either FAA (Section 4.2) or Carnoy (Section 4.3), transferred to 70% EtOH, were immersed in 1% Phospho-Tungstic Acid (PTA, Merck) in 70% EtOH for five days with daily refreshment. Samples washing and embedding in agarose in scanning tubes followed [84]. Scanning was performed with a 3D submicron imaging system (Xradia 510 Versa, Zeiss, Germany) with the following parameters: sealed transmission 30–160 kV, maximum source 10 W, lens magnitude 0.4×, acceleration 60 kV and 5 W or 80 kV and 7 W, exposure time 1 to 10 sec., and number of projections 1401 for normal resolution and 3201 for high resolution, the latter with pixel size 0.8 × 0.8 × 0.8 μm (see Supplementary Figure S3). 

The 3D reconstruction and analysis of the μCT scans were performed using Avizo v9.5 analysis software (Thermo Fisher Scientific, Waltham, MA, USA) as previously described [84]. Segmentation of vascular bundles and the pappus was performed with the segmentation feature. The following colour codes were assigned to the different floral organs and accompanying vascular bundles: green for pappus, red for petals, yellow for male structures (stamen including anthers), and pink for female structures (pistils including ovary, style, and stigma).

### 4.5. Primer Design and Verification 

Primer design was based on published *Taraxacum kok-saghyz* (*Tk*) sequences [62] homologous to lettuce (*Lactuca sativa*, *Ls*) and Arabidopsis genes of interest: the reference genes *UBIQUITIN CONJUGATING ENZYME*9 (*UBC*9) and *PROTEIN PHOSPHATASE*2A (*PP*2A) [61] and flower developmental genes *APETALA*1 (*AP*1)/*LsMADS*55 and *PISTILLATA* (*PI*)/*LsMADS*03 [53]. Information about used sequences, their GenBank IDs, *Tk*-homologous, and primers can be found in Supplementary Table S1. Usually, the two sequences most similar to Arabidopsis were chosen for primer design and more than one primer pair was made and tested, and the best performing set was for the final experiments. One of the primer sets, the one for *UBC*9, was designed over-intron, serving as a control for the presence of genomic (g)DNA residues in the cDNA, which turned out to be always negative. Primer verification was first done via a basic polymerase chain reaction (PCR) protocol [85] on DNA (see Section 4.6), using an anneal temperature of 60 °C, and second, via a two-step qPCR protocol (3 min. 95 °C; 45 × [15 s 95 °C − 1 min. 60 °C]; 5 min. 72 °C; ∞ at room temperature) on cDNA (see Section 4.6). Product lengths and intensity were checked on 2% agarose gels.

### 4.6. DNA Isolation, RNA Isolation, Samples Used, and cDNA Preparation

For the verification of qPCR primers (see Section 4.5), total DNAs were isolated from 5–20 mg tissues (leaf tips or buds) of a representative set of plants and tissues used in the qPCR experiments (described below). Tissues were placed in 2-mL round-bottom tubes containing two metal balls, on ice, and put into liquid N2. The frozen tissues were either grinded directly or after storage in −80 °C, at 30 rpm for 30 s two times in a mixer mill (MM 400, Retsch, Benelux) using pre-cooled (−20 °C) adapters. Total DNAs were isolated according to the cetyl-trimethyl-ammonium-bromide (CTAB) method [86], including the modifications for dandelion made by Vijverberg et al. [85]. Isolates were dissolved in Tris (10 mM)-EDTA (0.1 mM) to a final concentration of 100 ng/μL, and dilutions to 2 ng/μL in MQ made for (PCR) analysis and both were stored at −20 °C until use.

For RNA-isolations for qPCR, also 5–20 mg of deep-frozen tissues were used and grinded as described above. The tissues included whole young buds (FS; 0 = *stage 3–4*; Table 1), and either the upper floral part (F) and lower floral part (S), both including half of the beak, of older stages (1 = *stages 10–11*, closed bud; 2 = *stages 12–13*, open flower; 3 = *stage 14*, old flower, 3 days after pollination [DAP]; 7 = *stage 15*, 7 DAP, pappus only), separately, and a leaf (LF) sample. To separate the upper and lower floral parts, buds were cut in half after having the stem and most of the involucral bracts removed, then the florets were cut through the beaks using a sharp scalpel and a binocular and the remaining bracts and most of the inflorescence receptacle were removed; all quickly at room temperature. Biological triplicates, including three different plants of the same genotype, were processed for each stage and tissue. The analysis was based on one genotype, whereas some samples of other genotypes were analysed for confirmation. 

Total RNAs were isolated using TRIzol^®^ reagent (Ambion, Life technologies, 15596026), following the accompanying protocol, handling quickly on ice and under refrigerated conditions. After re-suspension in 100 µL diethyl-pyrocarbonate (DEPC) treated MQ, a second precipitation step was done to increase RNA purity. The final isolates were dissolved in 10–30 μL DEPC-MQ at a concentration of 100–200 ng RNA/μL and checked for 25S to 18S ribosomal peak quality and ratio ~2:1, e.g., by electrophoresis on 1.5% Agarose gel. Possible remaining DNAs were removed by treating 1.8 μg RNA in 22 μL with 1 Unit Turbo DNAse (Invitrogen/Ambion, AM2238) according to the manufacturer’s protocol. After this, the DNAse was inactivated by the addition of 2.5 µL 165 mM ethylene-diamine-tetra acetic acid (EDTA; final concentration of 15 mM) followed by 10 min. incubation at 65 °C. DNAse’s debris was removed by spinning for 10 min. at 2000 g, 4 °C, and pipetting 24.5 μL (1.6 μg RNA) of the upper layer to a new tube. RNA was then precipitated, resuspended in 16 μL DEPC-MQ, and the concentration was checked using a NanoDrop Spectrophotometer (~100 ng/μL). 

A total of 500 ng RNA in 9.5 μL was used for cDNA synthesis using the iScript kit (BioRad, 170–8890), in a total volume of 20 μL according to the manufacturers’ protocol. Then, the enzyme was inactivated at 95 °C for 1 min. and the cDNA diluted by adding 80 μL MQ and either used immediately for qPCR (see Section 4.7) or stored at −20 °C.

### 4.7. qPCR Analysis 

The qPCRs were performed at least twice, using iQ SYBR Green Supermix (BioRad, 170–8886, Life Science, Veenendaal, The Netherlands) and normal 96-wells PCR plates, in a qPCR machine (CFX96 BioRad). A mix of 4.8 μL MQ, 0.6 μL of each primer (10 μM), and 10 μL 2× iQ SYBR Green per sample was made on ice, of which 16 μL was pipetted per well and 4 μL cDNA (from Section 4.6) added. Plates were covered with Microseal ‘B’ plastic films (BioRad, MSB1001) and a two-step qPCR program was performed (see Section 4.5), followed by a melting curve analysis (5 s [65 to 95 °C] [ΔT +0.5 °C per cycle]). 

Calculations were performed per sample and results averaged over the biological triplicates at the end. Calculations included: (1) averaged Ct value per gene per sample over replicated qPCRs; (2) calculation of the ΔCt value by subtracting the Ct value of the reference gene from the Ct value of the sample; (3) obtaining the ΔΔCt value by subtracting the ΔCt value of the control sample from the ΔCt value of the sample; (4) calculating the differences in product copies as 2^ΔΔCt^; and (5) averaging the 2^ΔΔCt^ values of biological replicates and calculating the accompanying standard deviations. Since similar tissues of different developmental stages were analysed, true control samples (non-treated tissues) were lacking and a ΔCt value = 1 was used as control instead. This allowed comparison between genes in addition to comparison within genes. Results were depicted graph form using Excel.

## 5. Conclusions

Micro-morphological and molecular data provide ample evidence for the more generally accepted, but little investigated, hypothesis that the pappus in Asteraceae is homologous to the whorl of sepals. They show evidence of a pentamerous origin in dandelion, based on the stronger growth of the pappus parts located at the fusion zones between petals, while their alternipetalous positioning corresponds to the position where the sepals are expected. The formation of the pappus primordium ring, via the transition of cells from one to two rows in the outer cell layer, can be interpreted as the congenital fusion of the sepals. The distinct ring that forms later in the floret’s development shows a similar cell type, confirming its pappus nature. The pattern of floral A- and B-gene expression further supports the sepal identity of the pappus in dandelions. Our data also confirm that the petals initiate first, the stamens second, and the carpels third, in dandelion, as is common in Asteraceae. The pappus ring initiates at the base of the corolla and becomes visible at the moment that the stamens can be seen. This is heterochronic with respect to the sepals in other Angiosperms, which usually initiate first and the petals second, and homologous with respect to its location at the outside of the floret. We also visualize the composite nature of the dispersal-promoting structure in dandelions, which consists, apart from the pappus (whorl 1), of the beak (part of the fruit, whorl 4). These two organs also maturate differently, before and after anthesis, respectively. The composite, very efficient dispersal structure in dandelions can be considered highly derived. Together, our data support pappus–sepal homology and indicate that whorl 1 has been susceptible to change during evolution. They imply that whorl 1 organs and their underlying floral genes are potentially good candidates to investigate changes in response to rapidly changing environments.

The vasculature confirmed the absence of main bundles to the pappus but showed capillaries to supply the mature pappus parts instead. In addition, it illustrated that lateral petal bundles of neighbouring petals have been merged and are located in the zone between the fused petals in dandelion corolla tubes and ligules. This is opposite the stamen bundles, while the median petal bundles remained undetected in dandelion. This interesting characteristic is little mentioned, but appears to be unique to the Asteraceae and three other plant families within the Asteridae [78].

We also aimed to develop reference data for use in floral development studies and evolution studies in response to changing environments, with a focus on dispersal strategies. To this end, we provide floral developmental stage definitions of use in dandelion and other (related) Asteraceae (Table 1). We observed the presence of a different cell type along the outside of the carpellary tissue in young florets. We hypothesize that this layer represents the floret receptacle. This interpretation is supported by the expression of floral A-genes at the outside of the ovary in related species from the Asteraceae (lettuce and Gerbera), suggesting a receptacle origin of the inferior ovary in dandelion and other Asteraceae. Floret receptacle derived cells are probably also present along the beak in dandelions, possibly merged with some whorl 1 cells at the upper outside part of the beak to connect to the pappus. The main vascular bundles are located in the ovary wall, including the wall of the beak, as part of the floret receptacle.

In addition, we identified and verified the floral development genes *Tof-AP*1 and *Tof-PI* and the reference genes *Tof-UBC*9 and *Tof-PP*2A for qPCR analysis of various floral tissues, stages, and leaves in dandelion. The two reference genes were stably and comparably expressed across the different stages and tissues, being valuable for future expression studies in dandelions and possibly other Asteraceae. The expression patterns of the floral homologous A- and B-genes were as expected, supporting the utility of other floral homologous genes in dandelions. Further elucidation of the floral ABC(D)E-genes in dandelions will allow detailed analysis of specific tissues and stages and contribute to the unravelling of the whorl composition in the floral receptacle/beak region in dandelion florets. 

Together, our results confirm the sepal identity of the pappus and provide valuable reference data for future studies into dandelions and other Asteraceae.

## Figures and Tables

**Table 1:** Floral developmental stages of dandelion.

Stage		Stem (cm) *	Flower (mm) *	Pappus (mm) *	Beak (mm) *	Ovary (mm) *	Description	Figures Number	Samples Figure 7
**Floral organ initiation**	1	<0.1	<0.1			<0.1	flower primordia: domed (round) staged		
	2	<0.1	<0.1			<0.1	corolla primordia: ring/5 lobs	1B	FS0
	3	<0.1	0.1–0.2			<0.1	stamen primordia: 5 lobs; pappus primordia ring		FS0
	4	<0.1	0.2	0.01		<0.1	carpels initiated; organs elongating	2A; S1A	
	5	<0.1	0.3	0.05		<0.1	petals closed at top; carpels lower than anthers; pappus longer at petal fusion zones	2B, 4A,D; S1B	
	6	0.1	0.4	0.1		<0.1	floral part elongating; carpels passed anthers	4B,C	
**Floral maturation**	7	0.2	0.6	0.3	<0.1	0.1	ovary, beak and flower bottom pronouncing; pappus half corolla length; nectaries initiating	1C, 4E, 5A–C; S5A, SV1	
	8	0.5	2	1	0.05	0.2	ovary, beak, flower bottom, nectaries defined; vertical lines on fruit coat; scales on pappus	4F; S2	
	9	1–2	5	3	0.1	0.3	upper floral part quickly elongating; pigmentation of petals initiated	1D	
	10	3–6	8	5	0.2	0.5	upper floral part quickly elongating; horizontal lines on fruit coat	4G	F1, S1
	11	7–12	10	7	0.4	1	pigmentation of petals finalised; petals still closed at top; bud closed to loosen	1E	F1, S1
	12	13–20	17	8	0.7	1.5	anthesis: bud opens (D1); corolla’s opening; pollen mature; style enlarging; stigma closed	1F, 5D–F; S3,4,5B, SV2	F2, S2
**Seed formation**	13	21–30	25	9	1.5	2	also central florets open (D2,3–5); stigma lobes opening and receptive; ovary part elongating	1G	F2, S2
	14	>25	old	10	3	2.5	3 DAP: flower organs blown; beak and ovary elongating		F3, S3
	15	>25	blown	10	6	3	7 DAP: flower organs gone; beak elongating; fruit browning		F7, S7
	16	>25	blown	11	10	3.5	12 DAP: mature brown seed; long beak; pappus opening	1G-insert	

* Light and darker green indicates increasing and relatively fast growth of the particular organ, respectively; Sizes may vary somewhat with genotypes and growing conditions; D = Day; DAP = Days After Pollination.

## Data Availability

Not applicable.

## Supplementary Materials

The following material is available online at https://www.mdpi.com/article/10.3390/plants10081682/s1. Table S1: Primers used for qPCR in *Taraxacum officinale* floral tissues. Figure S1: Evidence for a pentamerous origin of the pappus in dandelion florets. Figure S2: Histological analysis of the floral whorls and the floret receptacle in dandelion florets. Figure S3: High resolution μCT scanning showing the capillaries to the pappus parts in a mature dandelion floret. Figure S4: Visualization of the nectaries in mature dandelion florets. Figure S5: Ovarian vasculature in μCT scans of a young and mature dandelion floret. Video SV1: 3D-model showing the entire vasculature of a young dandelion floret. Video SV2: 3D-model showing the entire vasculature of a mature dandelion floret.
