# Supplementary material for: Sepal Identity of the Pappus and Floral Organ Development in the Common Dandelion (Taraxacum officinale; Asteraceae)
Source: Plants (Basel). 2021 Aug 16;10(8):1682. doi: 10.3390/plants10081682 (PMC8398263; doi:10.3390/plants10081682)
Supplement: Supplementary file 1 [file plants-10-01682-s001.zip › plants-1314080-sup/plants-10-01682-TableS1,FiguresS1-S5.pdf]

plants

MDPI

Article

Sepal identity of the pappus and floral organ development in the common dandelion (*Taraxacum officinale*; Asteraceae)

Kitty Vijverberg [1,2,*], Monique Welten [1], Marjan Kraaij [3], Bertie J. van Heuven [1], Erik Smets [1], Barbara Gravendeel [1,2]

Supplementary data

Table S1. Primers used for qPCR in *Taraxacum officinale* floral tissues.

		**A-gene/*AP1*/ *LsMADS55* [1]**	**B-gene/*Pi*/ *LsMADS03* [1]**	***UBC9* [2]**	***PP2A* [2]**
**Arabidopsis**	**GenBank ID CDS**	NM_105581.1	NM_122031.1	AF325019.2	AY096543.1
	**GenBank ID gDNA**	At1g69120	At5g20240	At4g27960	At1g598300
	**Gene length (nt) [4]**	771	627	447	921
	**Exon number**	6	6	4	6
	**Exon lengths (nt)**	253, 115, 106, 83, 191, 173	253, 115, 106, 83, 191, 173	(89+)71, 125, 109, 142	253, 115, 106, 83, 191, 173
**Lactuca [1,2]**	**ID of used mRNA**	XM_023900141	XM_023906800	XM_023886937	XM_023905548
	**Gene Length (nt)**	762	591	446	921
	**Number of exons**	8	7	4	6
	**Exon lengths (nt)**	184, 77, 66, 99, 43,	188, 67, 63, 100, 30,	71, 130, 106, 139	253, 115, 106, 83,
	**Number of mRNAs**	4 (+5)	1	8	4
	**Coverage; Similarity mRNAs**	67%; 73-74%	54%; 74%	99%; 82-84%	96%; 80%
**Taraxacum [3]**	**ID of used gDNA**	utg432_03033	utg6773_25747	utg7189_17661	utg6374_12351
	**Number of exons & lengths**	idem lettuce	idem lettuce	idem lettuce	idem lettuce
	**Number of gDNAs**	1	2	6: 1, 1, 4	6: 1, 4, 1
**qPCR Primers**	**Primer name**	A-*AP*1-FW/RV [5]	B-*PI*-FW/RV	R-*UBC*9-FW/RV	R-*PP*2A-FW/RV
	**Primer location**	exon 1	exon 4	exon 2/3	exon 1
	**Primer length (nt)**	25; 23	27; 27	27; 28	22; 30
	**Tm Calculated (±0.2 ºC)**	60,0	60,8	60,5	59,8
	**Tm Used (ºC)**	60,0	60,0	60,0	60,0
	**Product length (nt)**	128	99	135 (gDNA 333)	115

**Primer name**	**3'--> 5' Sequence of primer**
A-*AP*1_FW [5]	GGGAGAGGAAAGGTACAACTTCGGA
A-*AP*1_RV [5]	GCAACTTCGGCATCACAGAGAAC
B-*PI*_FW	CACTTGGGAGGGGAAGACATAACATCT
B-*PI*_RV	CCTTTTTGACACGAATGTTTGTGAGCC
R-*UBC*9_FW	CAAGCAACAATTATGGGACCTCCAGAT
R-*UBC*9_RV	GATGTTTGGGTGGAAAACCTTTGTCCTG
R-*PP*2A_FW	CAACCGGTGAAGTGTCCGGTCA
R-*PP*2A_RV	CCATGAAGAGATAATTGGTATCAGGAGTAC

[1-3] References (see main paper): [1] [53]; [2] [61]; [3] *T. kok-saghyz*, [62]; [4] nt = nucleotides; [5] FW = forward; RV = reverse

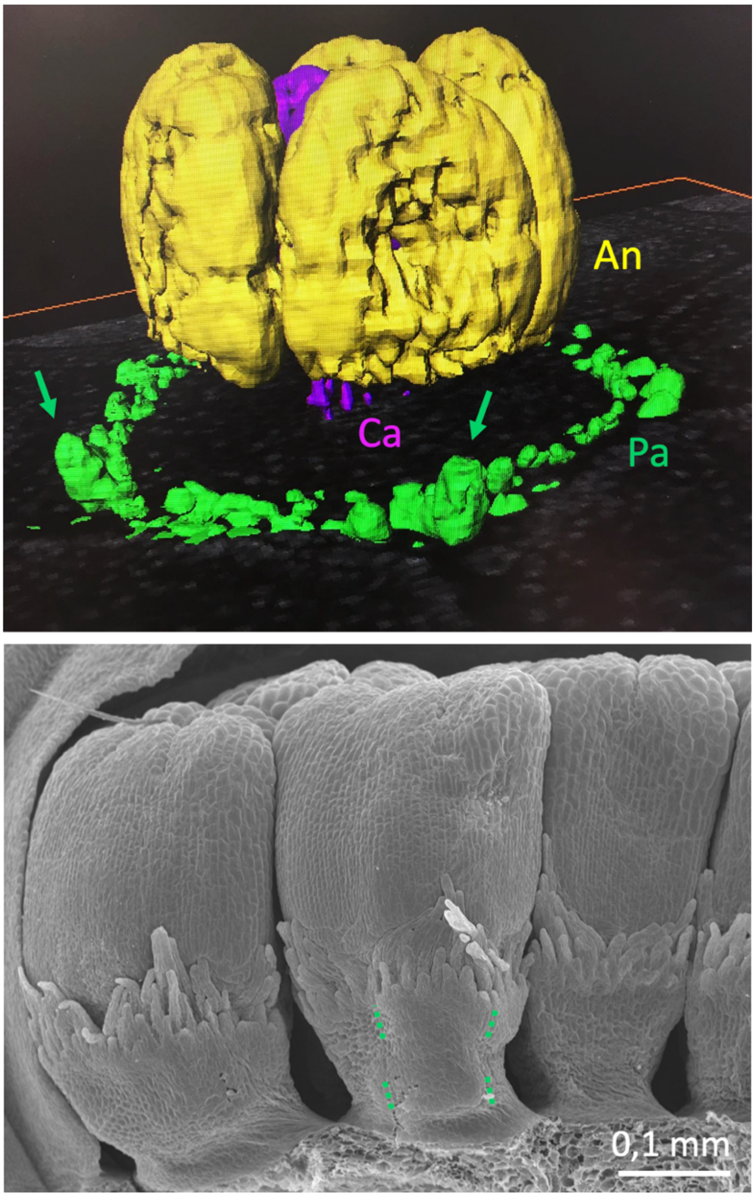

Figure S1. Evidence for a pentamerous origin of the pappus in dandelion florets. (**A**) A μCT scanning of a floret of *stage 4* (Table 1) with the pappus (Pa, green), anthers (An, yellow) and carpels (Ca; pink) segmented, showing that the pappus parts located at the petal fusion zones (green arrows), where the sepals are expected (Figure 3), are ahead in growth (the petals are not segmented). (**B**) SEM image of *stage 5* florets, with a weak indication for pappus development in five units (one indicated between green dotted lines). © Monique Welten and Bertie Joan van Heuven, respectively.

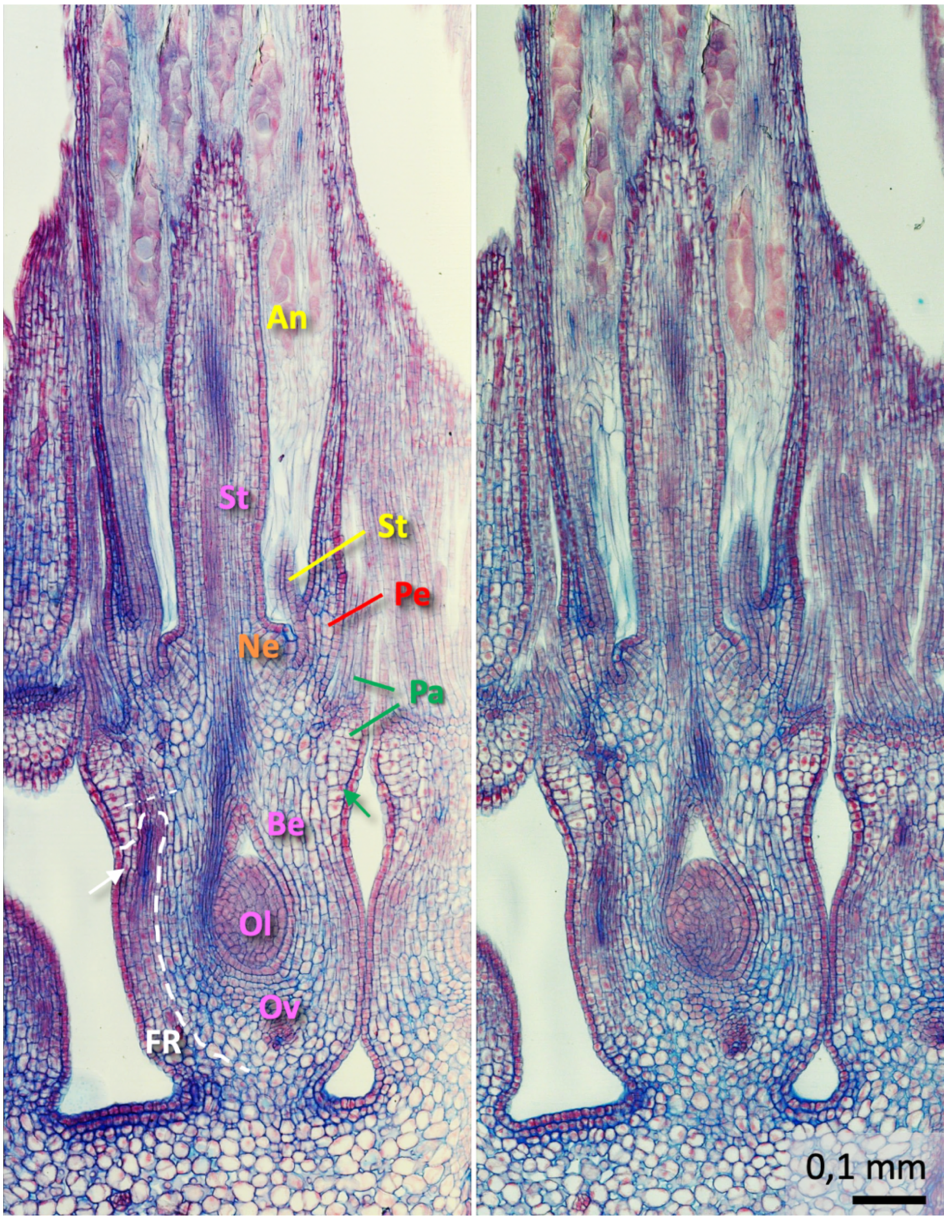

Figure S2. Histological analysis of the floral whorls and the floret receptacle in dandelion florets. Two consecutive vertical sections of a *stage 8* floret (Table 1), showing the transition from one to two cells at the base of the pappus (Pa, green arrow), interpreted as the congenital fusion of the sepals (see also Figure 2). Together with the similar cell type of the cells in this ring, this supports that the entire ring is a modified calyx and whorl 1. The individual pappus parts arise on this ring. Additionally, the sections show the presence of a layer of a different cell type around the ovary (Ov; indicated with a white dotted line). This was also clearly present in younger florets (Figure 2A) and we hypothesize that these cells belong to the Floret Receptacle (FR). This separation between the ovary (whorl 4) and receptacle was supported by expression studies in lettuce (see main text, Section 3.3). The beak (Be) is part of the ovary (whorl 4) and the receptacle tissue probably continues along its outside (supported in older stages; not shown), while the top of the beak wall may include some whorl 1 tissue in addition (white arrow), probably to connect to the pappus. According to this interpretation, the vascular bundles (dark cells near white arrow) are located in the floret receptacle, along the ovary and beak, which is supported by μCT-scanning results (see Supplementary Figure 4B). Pe (red) = petals; St / An (yellow) = stamen including the anthers; Ov, Ol, Be, St (pink) = ovary, ovule, beak, style; Ne (orange) = nectaries. Stain: Etzold. © Marjan Kraaij.

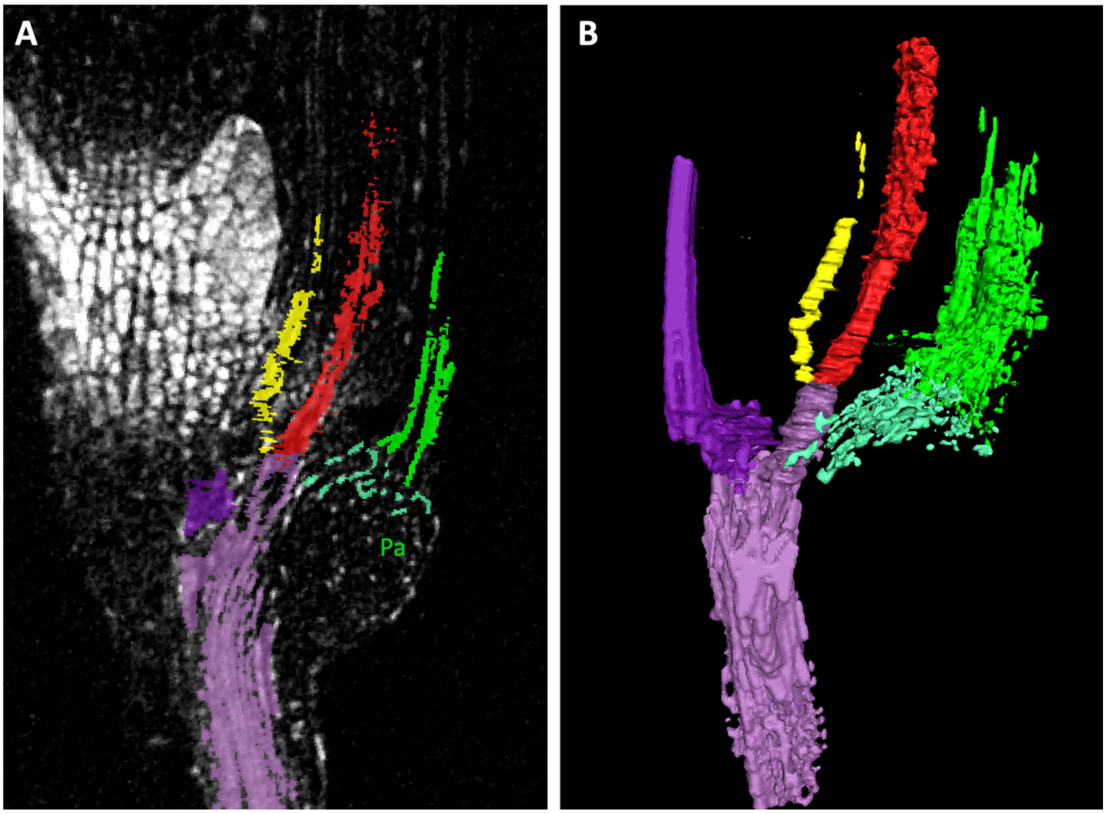

Figure S3. High resolution µCT scanning showing the capillaries to the pappus parts in a mature dandelion floret. Segmentation of part of the vasculature in a dandelion floret of *stage 12* (Table 1). (**A**) A vertical section, showing the capillaries (green 1) to the pappus parts (green 2) in the upper part of pappus ring (Pa); (red) = petal bundle; (yellow) = stamen bundle; (pink 1) = carpel bundle; (pink 2) = main bundle. (**B**) The same bundles as in A after high resolution scanning, showing the capillaries to the pappus to branch off from the main bundle just above and around the split to the carpels and well-below the split to the petals and stamen (see also Figure 6C). © Monique Welten

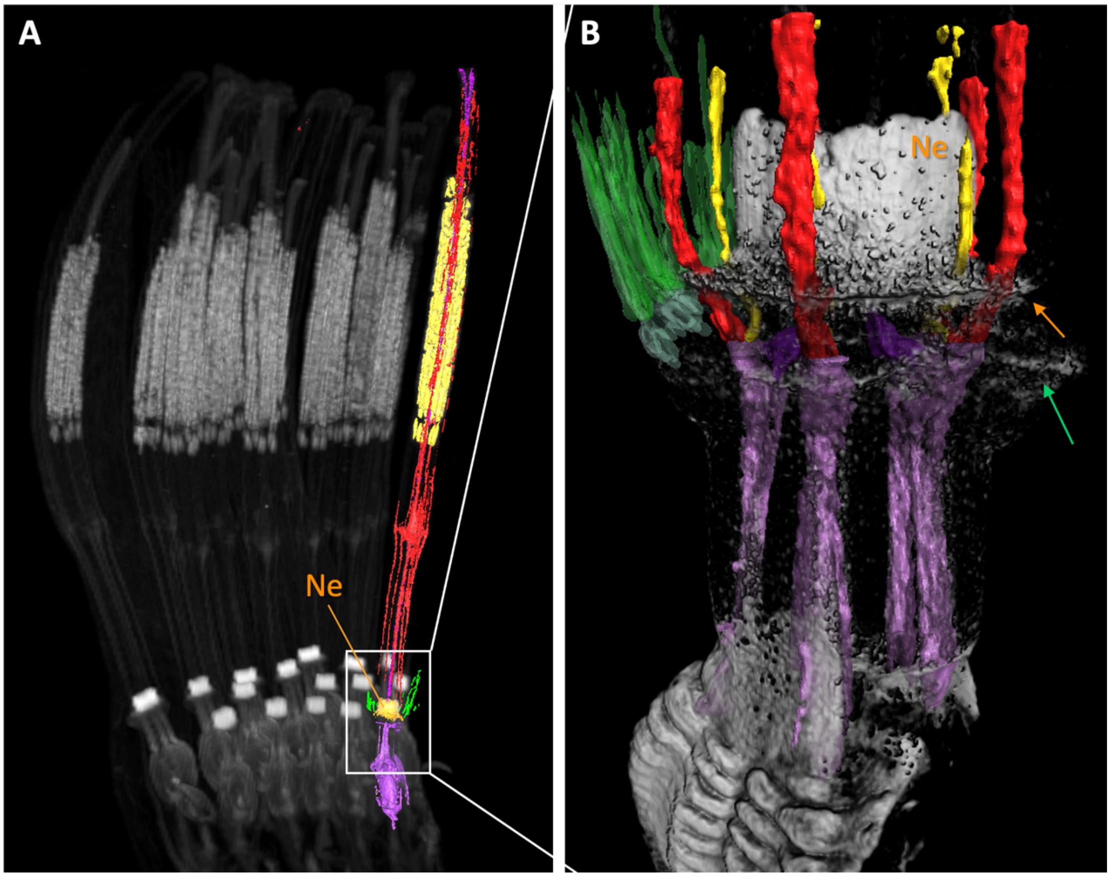

Figure S4. Visualization of the nectaries in mature dandelion florets. (**A**) A µCT scanning showing part of a dandelion capitulum just before anthesis (*stage 12,* Table 1), with all *organs* of one floret segmented and the others in grey-white background. The necatries (Ne, orange; white densely celled if unsegmented) are located on top of the ovary (pink) in dandelions, encircled by the pappus (green), petals (red) and stamen (yellow). The model clearly shows the thickening in the corolla (red; at c. 1/3rd), where it unfolds into a ligule, and the cell-dense anthers (yellow), arranged in a tube. Additionally, the style and stigmas (pink) can be seen, that have just passed the anther tube and start to open. (**B**) Enlargement of one of the florets shown in A, with the *vasculature* segmented (explained in Figures 5D-F), and some pappus parts in addition (green). The model clearly shows the cell dense nectaries and two rings, one that represents the pappus ring / the-calyx (green arrow) and the second that is part of the nectaries and connects to the petal and anther bundles (orange arrow), supposedly for their supply. © Monique Welten

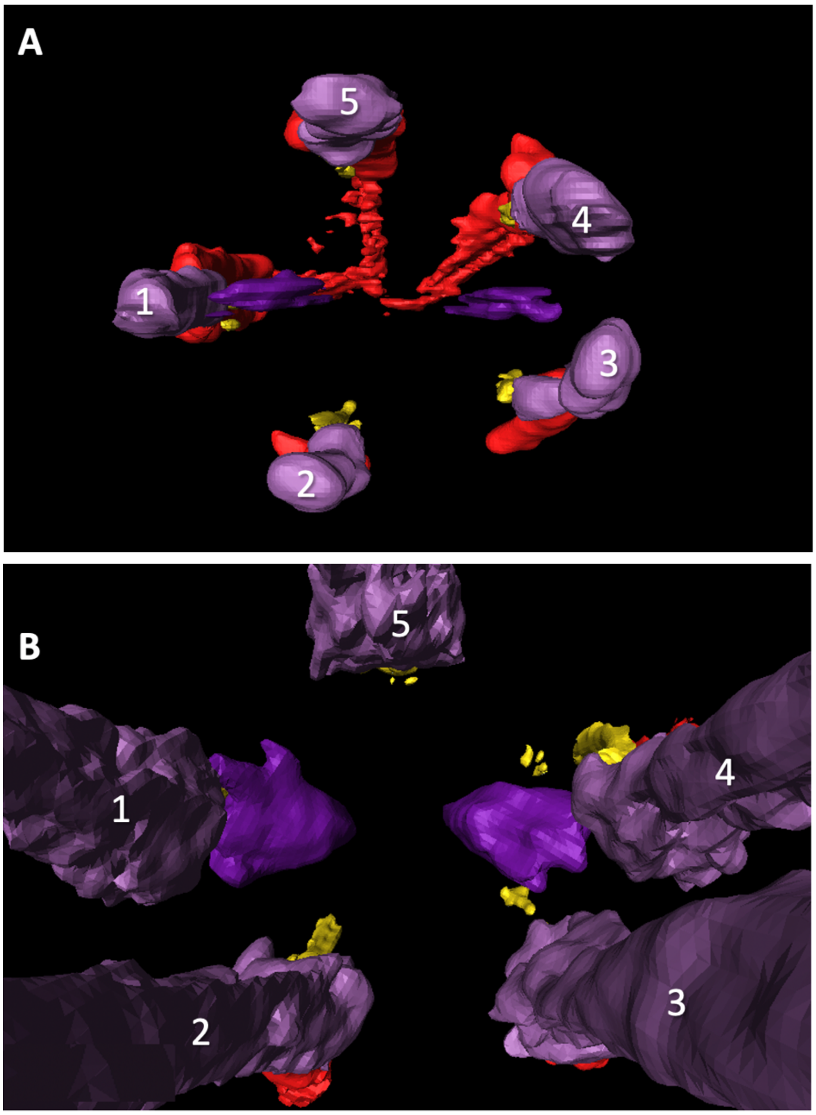

Figure S5. Ovarian vasculature in µCT scans of a young and mature dandelion floret. Viewing direction is from below (the ovary-side); numbers denote the main bundles (pink 1); (pink 2, bright) = carpel bundles; (red) = petal bundles; (yellow) = stamen bundles. (A) Floret of *stage 7* (Table 1) showing the splits of bundle 1 and 3 to the carpels, with the latter moved to the centre between bundles 3 and 4 (as in the diagram in Figure 6B). (**B**) Floret of *stage 12,* visualizing the same. © Monique Welten

Supplementary Video V1. 3D-model showing the entire vasculature of a young dandelion floret. The movie corresponds to the floret of *stage 7* (Table 1) shown and described in Figure 5A-C. It shows the five main bundles (pink 1), the branching off of bundles to the carpels (pink 2), and the five bifurcations each to a petal (red) and a stamen/anther (yellow). The model also shows the bifurcation of one of the five petal bundles (at the front at 0 sec.), where the corolla forms the ligule. In the second half of the movie the pappus (green transparent) and anthers (yellow transparent) are shown in addition. (© Monique Welten

Supplementary Video V2. 3D-model showing the entire vasculature of a mature dandelion floret. The movie corresponds to the floret of *stage 12* (Table 1) shown and described in Figure 5D-F. It shows the five main bundles (pink 1), the branching off of bundles to the carpels (pink 2), and the five bifurcations each to a petal (red) and a stamen/anther (yellow). After five seconds, part of the capillaries (green 1) to the pappus parts, and some pappus parts (green 2, transparent) are shown in addition, and after twelve seconds, also the nectaries (orange-transparent) become visible. The bifurcation of one of the five petal bundles cannot be seen in this movie, since it concerns the lower part of the corolla, where it still forms a tube. © Monique Welten
